# Rational Development of Hypervalent Glycan Shield‐Binding Nanoparticles with Broad‐Spectrum Inhibition against Fatal Viruses Including SARS‐CoV‐2 Variants

**DOI:** 10.1002/advs.202202689

**Published:** 2022-11-15

**Authors:** Ying Li, Shuxin Xu, Qing Ye, Hang Chi, Zhanchen Guo, Jingran Chen, Mei Wu, Baochao Fan, Bin Li, Cheng‐Feng Qin, Zhen Liu

**Affiliations:** ^1^ State Key Laboratory of Analytical Chemistry for Life Science School of Chemistry and Chemical Engineering Nanjing University Nanjing 210023 P. R. China; ^2^ Department of Virology State Key Laboratory of Pathogen and Biosecurity Beijing Institute of Microbiology and Epidemiology AMMS Beijing 100071 P. R. China; ^3^ Institute of Veterinary Medicine Jiangsu Academy of Agricultural Sciences, Key Laboratory of Veterinary Biological Engineering and Technology, Ministry of Agriculture Nanjing 210014 P. R. China

**Keywords:** broad‐spectrum antivirals, glycan shields, molecular imprinting, nanoparticles, severe acute respiratory syndrome coronavirus 2

## Abstract

Infectious virus diseases, particularly coronavirus disease 2019, have posed a severe threat to public health, whereas the developed therapeutic and prophylactic strategies are seriously challenged by viral evolution and mutation. Therefore, broad‐spectrum inhibitors of viruses are highly demanded. Herein, an unprecedented antiviral strategy is reported, targeting the viral glycan shields with hypervalent mannose‐binding nanoparticles. The nanoparticles exhibit a unique double‐punch mechanism, being capable of not only blocking the virus–receptor interaction but also inducing viral aggregation, thereby allowing for inhibiting the virus entry and facilitating the phagocytosis of viruses. The nanoparticles exhibit potent and broad‐spectrum antiviral efficacy to multiple pseudoviruses, including severe acute respiratory syndrome coronavirus 2 (SARS‐CoV‐2) and its major variants (D614G, N501Y, N439K, Δ69‐70, Delta, and Omicron; lentiviruses expressing only the spike proteins), as well as other vital viruses (human immunodeficiency virus 1 and Lassa virus), with apparent EC50 values around the 10^−9^ m level. Significantly, the broad‐spectrum inhibition of authentic viruses of both wild‐type SARS‐CoV‐2 and Delta variants is confirmed. Therefore, this hypervalent glycan‐shield targeting strategy opens new access to broad‐spectrum viral inhibition.

## Graphical abstract

The hypervalent mannose‐binding nanoparticles, targeting the viral glycan shields, are rationally developed by controllable molecular imprinting technique. The nanoparticles could effectively block the virus–receptor interaction and induce viral aggregation, facilitating immune clearance. The nanoparticles harbor broad‐spectrum antiviral efficacy to severe acute respiratory syndrome coronavirus 2 and its major variants, as well as other vital viruses.

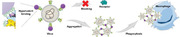

## 1 Introduction

Infectious diseases caused by viruses, including human immunodeficiency virus 1 (HIV‐1), influenza, hepatitis C virus (HCV), Zika, and Ebola, have been threatening human beings profoundly.^[^ ^1^ ^]^ In addition to these established diseases, new infectious diseases intermittently emerge. Notably, the newest outbreak of coronavirus disease 2019 (COVID‐19) has posed a severe threat to global public health and social development.^[^ ^2^ ^]^ Though therapeutic and prophylactic strategies, including vaccines,^[^ ^3^ ^]^ neutralizing antibodies,^[^ ^4^, ^5^ ^]^ and small molecular drugs,^[^ ^6^ ^]^ have been developed, the rapidly changing antigenic profiles of viruses present notable challenges. Indeed, the constantly emerging variants of the severe acute respiratory syndrome coronavirus 2 (SARS‐CoV‐2) have raised concerns about resistance to the current therapeutics or vaccines.^[^ ^7^, ^8^, ^9^ ^]^ Particularly, the newest variants of concern (VOC) Omicron (B1.1.529), due to the presence of more than 30 mutations in the spike, has been disclosed to be remarkably resistant to many authorized antibody drugs,^[^ ^9^ ^]^ resulting in reinfection and vaccine breakthroughs. Therefore, broad‐spectrum inhibitors of pathogenic viruses, especially various mutated strains of SARS‐CoV‐2, are highly demanded.

Glycosylation is a universal post‐translational modification, which is responsible for multiple crucial biological roles.^[^ ^10^ ^]^ Numerous viruses, including HIV‐1, SARS, influenza, Lassa, Zika, and Ebola, have evolved to utilize host‐cell machinery to modify their proteins with “self” glycans during replication, resulting in extensively glycosylated envelope proteins.^[^ ^11^ ^]^ The host cell‐derived glycans play critical roles during the viral life cycle. Of note, extensively glycosylated viral proteins facilitate escaping from immune surveillance of infected hosts by shielding the immunogenic protein surface with a dense coat of host‐derived glycans.^[^ ^12^, ^13^, ^14^ ^]^ In particular, many viral glycoproteins do not follow the classical secretion pathway, but bud directly from the endoplasmic reticulum and shift to the plasma membrane, bypassing further complex and diverse glycosylation in the Golgi apparatus, which yields viral populations that are occupied by considerable high‐mannose glycans.^[^ ^15^, ^16^, ^17^, ^18^ ^]^ Furthermore, these oligomannose glycan‐containing viral glycoproteins can function as host‐cell attachment factors to enhance or facilitate the infection of target cells.^[^ ^11^ ^]^ Similarly, SARS‐CoV‐2 is decorated by a great quantity of highly glycosylated proteins, which extensively affects host recognition, binding, penetration, recycling, and pathogenesis.^[^ ^19^ ^]^ The glycans on N234 and N709 of the SARS‐CoV‐2 spike are mainly high‐mannose glycans, while the other six positions, including N61, N122, N603, N717, N801, and N1074, are modified with a mixture of high‐mannose and complex glycans.^[^ ^20^, ^21^ ^]^ Totally, 28% of N‐linked glycan compositions observed on the SARS‐CoV‐2 spike are oligomannose glycans.^[^ ^21^ ^]^ Therefore, the conserved structural features (plentiful high mannose) of the glycans could be a breakthrough point to deal with constantly mutating viruses and achieve broad‐spectrum inhibition.

Due to the weak immunogenicity and low accessibility, targeting reagents specific to glycans of viruses are extremely rare.^[^ ^22^ ^]^ The reported affinity reagents specific to high‐mannose are limited to a few antibodies and lectins.^[^ ^23^, ^24^ ^]^ Among them, monoclonal antibody 2G12, which can bind high‐mannose glycans on HIV‐1 envelope protein GP120, has exhibited a broad‐spectrum inhibition against HIV‐1.^[^ ^23^ ^]^ However, 2G12 failed to block the viral entry of SARS‐CoV‐2.^[^ ^25^ ^]^ Besides, a variety of mannose‐specific lectins like *allium porrum* agglutinin, *hippeastrum hybrid* agglutinin, griffithisin, and cyanovirin‐N exhibit antiviral activities against many coronaviruses. However, the application potential of lectins was limited due to some unfavorable responses like immunogenicity, mitogenicity, and inflammatory activity,^[^ ^26^ ^]^ and the multivalent mannose glycoderivatives, as virus mimics, also exhibit blocking efficacy for certain viruses, but they target host cells rather than viral glycans.^[^ ^27^, ^28^ ^]^ Therefore, how to achieve broad‐spectrum virus inhibition by targeting the glycan shields remains a significant challenge.

Molecularly imprinted polymers (MIPs), which are also referred to as artificial or plastic antibodies, are chemically synthesized receptors with antibodies‐mimicking binding properties via copolymerization in the presence of templates.^[^ ^29^, ^30^ ^]^ Due to their advantages over biological antibodies, such as ease of preparation, cost efficiency, and storage stability,^[^ ^29^, ^30^, ^31^, ^32^, ^33^, ^34^, ^35^, ^36^ ^]^ MIPs have exhibited great potential in a range of promising applications, for instance, disease diagnosis,^[^ ^31^ ^]^ cancer therapy,^[^ ^32^ ^]^ toxin neutralization,^[^ ^33^ ^]^ and virus recognition.^[^ ^34^ ^]^ Although MIPs against viruses have been developed,^[^ ^35^, ^36^ ^]^ MIPs with broad‐spectrum inhibitory efficacy have not been reported yet.

**Scheme 1:**
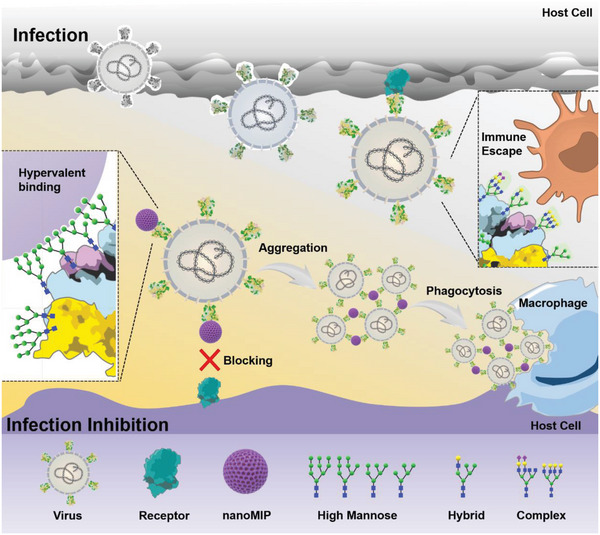
Illustration of virus inhibition by anti‐high mannose nanoMIP.

Herein, we report the rational development of glycan shield‐binding molecularly imprinted nanoparticles (nanoMIP) with potent and broad‐spectrum inhibitory efficacy to high‐mannose‐carrying viruses, especially to SARS‐CoV‐2 and its variants. The nanoMIP was prepared by integrating our unique strengths in boronate affinity‐based saccharide recognition,^[^ ^37^, ^38^, ^39^ ^]^ as well as a recently reported controllable molecular imprinting technique.^[^ ^40^ ^]^ As illustrated in **Scheme** **1**, harboring hypervalent binding capability to mannose (>2500 binding sites for mannose), the nanoMIP could specifically bind high mannose glycans—a conservative structural feature of the glycan shields of many highly mutating viruses with high avidity. Through strongly binding glycan shields of virions, the nanoMIP could effectively block the virus–receptor interaction. Beyond this, the hypervalency of nanoMIP could induce viral aggregation, facilitating immune clearance and further blocking the virus entry to host cells. The nanoMIP was experimentally demonstrated, at the pseudovirus level, to harbor the broad‐spectrum antiviral efficacy to SARS‐CoV‐2 variants, including D614G, N501Y, N439K, Δ69‐70, Delta, and Omicron strains, as well as other vital viruses with glycan shields including HIV‐1 and LASV. Importantly, the high anti‐virus potency of nanoMIP was verified with the EC50 values toward all tested viruses around 10^−9^ m. The broad‐spectrum neutralization efficacy to authentic viruses was confirmed by both wild‐type and Delta SARS‐CoV‐2 viruses. Thus, this study first provided a virus inhibitor toward different species of viruses, holding great potential to be expanded for the inhibition of newly emerging variants of SARS‐CoV‐2 and other fatal viruses in the future.

## 2 Results

### 2.1 Preparation and Characterization of NanoMIP

**Figure 1:**
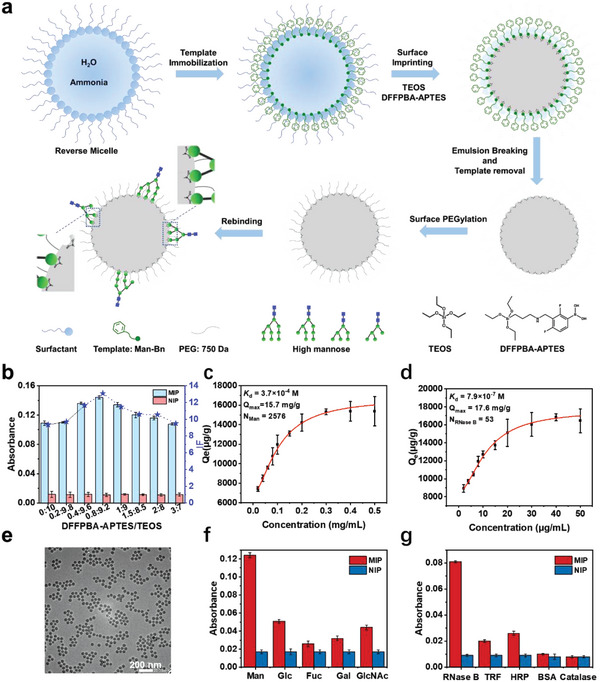
Design and characterization of nanoMIP. a) Preparation procedure of nanoMIP. b) Optimization of imprinting conditions. c,d) Binding isotherms and affinity measurement of nanoMIP at the monosaccharide and protein level, respectively. e) Representative TEM image of nanoMIP. f,g) Selectivity test, comparison of the UV absorbance for test monosaccharides (at 230 nm) and proteins (at 214 nm) captured by nanoMIP and corresponding NIP. Mean ± SD, *n* = 3.

The nanoMIP was synthesized according to a recent approach called reverse microemulsion‐confined epitope‐oriented surface imprinting and cladding (ROSIC)^[^ ^40^ ^]^ with major modifications. The preparation procedure is schematically illustrated in **Figure** **1**a. Briefly, a reverse microemulsion formed with an appropriate surfactant (Triton X‐100) and an oil phase (cyclohexane) was first constructed as a nanoreactor to confine the polymerization in an aqueous phase. For efficient mannose imprinting, an amphiphilic molecule 2‐benzyl‐*α*‐D‐mannopyranoside (Man‐Bn) was designed and synthesized as the imprinting template (Figure S1, Supporting Information). Due to the presence of a hydrophobic benzyl group, Man‐Bn was anchored at the interface between the aqueous phase and the oil phase, with the imprinting mannose part protruding into the confined aqueous phase. The monomers used for the imprinting were well chosen. Here, tetraethyl orthosilicate (TEOS) was chosen as a monomer to form a silica skeleton. Moreover, 3‐aminopropyltriethoxysilane modified with 2,4‐difluoro‐3‐formylphenylboronic acid (DFFPBA‐APTES) was synthesized as another monomer, which provides a covalent force to bind mannose via the interaction between the boric group of the monomer and the cis‐dihydroxy group on mannose. After the polymerization of monomers, the Man‐imprinted nanoparticles were released from the microemulsion and the template was extracted, leaving well‐formed imprinted cavities complementary to mannose in aspects of shape, size, and functionalities. Furthermore, in order to improve biocompatibility and stability, the Man‐imprinted nanoparticles were cladded with polyethylene glycol (PEG). In comparison, non‐imprinted nanoparticles (NIP) were also prepared correspondingly with the same procedure except for the absence of a template.

The prepared nanoMIP was thoroughly optimized by changing the monomeric ratio, the amount of template used, and the chain length of PEG. The monomer ratio was optimized in terms of imprinting factor (IF), which is determined by the ratio of mannose amount captured by Man‐imprinted nanoparticles over that by NIP. As shown in Figure 1b, the imprinting at the ratio of 0.8:9.2 (DFFPBA‐APTES: TEOS) gave the best IF value (13.1), which was outstanding in the imprinting of saccharides. Thus, this monomer ratio was chosen for further characterization. Subsequently, the amount of Man‐Bn template used was optimized. Scatchard plots for the binding of Man‐imprinted nanoparticles toward mannose were drawn and the binding sites were calculated (see “Estimation of binding valency and apparent molecular mass of nanoMIP” in Experimental Section). As shown in Figure 1c,d and Figure S3, Supporting Information, compared with ≈60 mannose molecules bound by each nanoparticle prepared with 1 mg template, Man‐imprinted nanoparticles prepared with 100 mg templates bond ≈2576 mannose moieties for each. To the best of our knowledge, such hypervalent binding has never been reported before, which provides a unique strength for glycan shield binding. As estimated by Chem3D, the distance between different terminal mannoses of high‐mannose glycans ranges from 13.8 to 15.6 Å (Figure S5, Supporting Information). It is indicated that the spacing between 2576 mannose‐binding sites on a 40‐nm Man‐imprinted nanoparticle is small enough to recognize different terminal mannoses of high‐mannose glycans simultaneously, bringing in high binding avidity to high‐mannose glycans. Besides, the multivalent binding capability to high‐mannose was also displayed by the binding with 53 RNase B (high mannose type) molecules on single nanoparticles (Figure 1d). Man‐imprinted nanoparticles with more template (1 g) were also prepared. However, the microemulsion was unstable, and extremely uneven particles were observed (Figure S4, Supporting Information), Therefore, Man‐imprinted nanoparticles prepared with a 100 mg template, which could well balance the imprinted cavities and microemulsion stability, were employed for further use. Scatchard plot analysis gave a dissociation constant (*K*_d_) of 3.7 × 10^−4^ m to mannose and 7.9 × 10^−7^ m to RNase B, verifying the superior binding capability of Man‐imprinted nanoparticles toward mannose and high‐mannose glycans. Furthermore, the length of the PEG chain was optimized in terms of the IF value, as well as the stability of prepared PEGylated Man‐imprinted nanoparticles. As shown in Figures S6, S7, and S30, Supporting Information, the dispersions of Man‐imprinted nanoparticles modified with PEG750 exhibited the best stability and gave the overall best IF values. Therefore, PEG750 was chosen for further investigations.

The resulting nanoMIP was characterized using transmission electron microscopy (TEM) and scanning electron microscopy (SEM). As shown in Figure 1e and Figure S9, Supporting Information, monodispersed nanoMIP with well‐defined spherical morphology was obtained, and the mean diameter was 39.5 ± 2.3 nm (Figure S8, Supporting Information). Additionally, some basic characterization of nanoMIP, such as Fourier transform infrared (FT‐IR) spectrum, X‐ray photoelectron spectroscopy (XPS), and energy dispersive spectroscopy (EDS) mapping, are shown in Figures S10, S12, and S13, Supporting Information. Furthermore, the specificity of nanoMIP was evaluated at the monosaccharide and protein levels, respectively. As shown in Figure 1f,g, the specific adsorption of mannose by the nanoMIP was significantly higher than that of other interfering monosaccharides (Figure 1f). Notably, the recognition performance of nanoMIP is superior to some mannose‐binding lectins (MBLs) such as mannose‐binding *Morus nigra* agglutinin and *Artocarpus integrifolia* agglutinin.^[^ ^41^ ^]^ As shown in Figure 1g, the specific binding to RNase B by nanoMIP was almost four times higher than that of interfering proteins modified with complex glycans including transferrin (TRF) and horseradish peroxidase (HRP). Additionally, the nanoMIP showed little‐to‐no specific binding to non‐glycosylated proteins including bovine albumin (BSA) and catalase. Considering that plasma glycoproteins are mainly modified with complex glycans as revealed by mass spectrometry,^[^ ^42^ ^]^ the specificity of the nanoMIP described above is well accepted, holding good potential for systemic or local administration. All the above characterization results verified that nanoMIP capable of binding multiple high‐mannose glycans (more than 50) on single nanoparticles have been successfully prepared.

### 2.2 NanoMIP as Artificial Antibody to High‐Mannose Glycans

**Figure 2:**
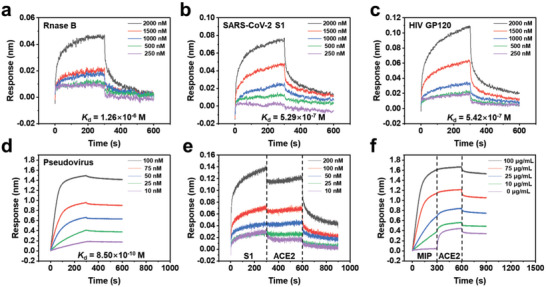
Binding affinity and competitive binding of nanoMIP by biolayer interferometry. a–c) The binding curves of nanoMIP toward RNase B, SARS‐CoV‐2 S1, and HIV‐1 GP120 protein, respectively. The nanoMIP was loaded onto APS biosensors. d) The binding curves of nanoMIP toward SARS‐CoV‐2 pseudovirus, with pseudovirus loaded on APS biosensors. e) The competitive binding curves of nanoMIP at the protein level, with nanoMIP loaded on APS biosensors. f) The competitive binding curves of nanoMIP at the pseudovirus level, with pseudovirus loaded on APS biosensors.

To verify the feasibility of nanoMIP as the artificial antibody against high‐mannose glycans, the binding affinity and kinetics of different proteins modified with high‐mannose glycans were investigated by biolayer interferometry (BLI). First of all, RNase B, containing only one site glycosylated with high‐mannose glycans, was used as a target protein. The *K*_d_ value was determined to be 1.3 × 10^−6^ m (**Figure** **2**a). Compared with that to mannose, the *K*_d_ value was improved by 2–3 orders of magnitude, which was attributed to the hypervalency of nanoMIP. Considering several N‐linked glycosylation sites on the SARS‐CoV‐2 spike are occupied with high‐mannose glycans,^[^ ^21^ ^]^ the binding to SARS‐CoV‐2 S1 protein was studied. As shown in Figure 2b, the *K*_d_ value was calculated to be 5.3 × 10^−7^ m, which is about one order of magnitude lower than that of RNase B due to the increase of high‐mannose sites on S1 protein. In comparison, no obvious binding of the NIP to SARS‐CoV‐2 S1 protein or RNase B was observed (Figure S14, Supporting Information). Besides, the binding of the nanoMIP to the HIV‐1‐envelope (Env) was also studied. The HIV‐1‐Env, as one of the most densely glycosylated viral proteins, exhibits about 60% oligomannose glycans.^[^ ^16^ ^]^ The *K*_d_ value was found to be 5.4 × 10^−7^ m (Figure 2c). Based on the above results, it is clear that the nanoMIP could serve as an efficient artificial antibody to high‐mannose glycans with sufficient avidity.

Considering 28% of N‐linked glycan compositions observed on the SARS‐CoV‐2 spike are oligomannose glycans,^[^ ^21^ ^]^ we further verified the target binding capability of nanoMIP to SARS‐CoV‐2 pseudovirus by BLI. The pseudovirus was loaded onto APS biosensors, while the nanoMIP of different concentrations was used as the ligand. The *K*_d_ value was measured to be 8.5 × 10^−10^ m (Figure 2d), which is three orders of magnitude lower than that of the SARS‐CoV‐2 S1 protein. The dramatically enhanced binding strength suggested that the nanoMIP could bind virions with high avidity toward glycan shields, which was further verified by negative controls (Figure S15, Supporting Information). We then explored the competitive binding of the nanoMIP with the SARS‐CoV‐2 receptor angiotensin converting enzyme 2 (ACE2). The competitive binding of nanoMIP with ACE2 was first evaluated at the protein level. The nanoMIP was loaded on APS biosensors. As shown in Figure 2e, no obvious binding of ACE2 was observed to SARS‐CoV‐2 S1 pre‐binding nanoMIP even when the concentration of ACE2 was up to 200 nm. This suggests the blockade effect of nanoMIP on the binding of SARS‐CoV‐2 S1 and ACE2. Furthermore, the competition assay was evaluated at the virus level. SARS‐CoV‐2 pseudovirus was first loaded onto biosensors and then bound with nanoMIP of different concentrations, followed by competition with ACE2. As shown in Figure 2f, as increasing the concentration of nanoMIP, the binding with ACE2 decreased. At a concentration of 100 µg mL^−1^, the nanoMIP completely blocked the binding of ACE2 to pseudovirus. Such excellent blocking performance might be attributed to two aspects: 1) Steric hindrance. The SARS‐CoV‐2 virion is a nanosized sphere around 100 nm and spike trimers are about 20 nm in length,^[^ ^43^ ^]^ while nanoMIP is around 40 nm. Thus, nanoMIP may have posed a strong steric hindrance to ACE2 approaching virion. 2) Conformational changes of the spike. Considering that the flexible 2G12 (high mannose‐binding antibody)^[^ ^23^ ^]^ and high‐mannose binding aptamer^[^ ^44^ ^]^ could bind the S protein but failed to block ACE2 binding, we speculate that the potent blocking effect of nanoMIP may have benefited from its structural rigidity and multivalent binding capacity, which may have induced the conformational changes^[^ ^45^ ^]^ of the S protein and then significantly affected the binding of ACE2.

### 2.3 Broad‐Spectrum Inhibition of Viruses via Anti‐Glycan Shields

**Figure 3:**
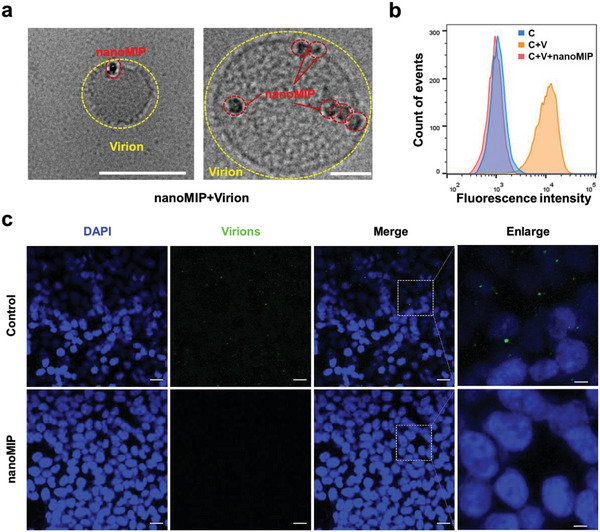
Inhibition of SARS‐CoV‐2 pseudovirus (lentiviral pseudoparticles) attachment to ACE2‐HEK293T cells by nanoMIP. a) Cryo‐transmission electron microscopy images for the SARS‐CoV‐2 pseudovirus particle binding with nanoMIP (25 µg mL^−1^). Scale bars, 200 nm (left) and 50 nm (right). b) Flow cytometry for the pseudovirus attachment to ACE2‐HEK293T cells (C, control group; C + V, cells incubated with pseudovirus; C + V + MIP, cells incubated with pseudovirus pretreated with nanoMIP; 1 mg mL^−1^ nanoMIP was used). c) Confocal fluorescence images for the pseudovirus attachment to ACE2‐HEK293T cells in the presence of nanoMIP (1 mg mL^−1^). Green represents the virions stained by DIO and blue represents the nucleus stained by DAPI. Scale bars, 20 µm (5 µm for enlarged images).

Since the nanoMIP exhibited an effective blocking effect on the binding between SARS‐CoV‐2 and ACE2, the inhibitory efficacy of the nanoMIP to SARS‐CoV‐2 infection was further investigated. Before virus neutralization assays, the complex of nanoMIP and SARS‐CoV‐2 pseudovirus (lentiviral pseudoparticle) was visualized by cryo‐transmission electron microscopy (Cryo‐TEM). As shown in **Figure** **3**a and Figure S17, Supporting Information, binding of multiple nanoMIP particles with one SARS‐CoV‐2 pseudovirus virion was observed, and no obvious binding was observed for NIP control (Figure S18, Supporting Information). Moreover, the blockade efficacy of nanoMIP was also investigated at the cellular level. After being pretreated with nanoMIP, little‐to‐no virus particles stained by 3,3‐dioctadecyloxacarbocyanine perchlorate (DiO) were detected on the surface of HEK293T‐ACE2 cells by flow cytometry (Figure 3b) and confocal fluorescence microscopy (Figure 3c), compared with DIO‐only control (Figure S31, Supporting Information) and pseudovirus without glycans (Figure S32, Supporting Information). This indicates that the nanoMIP could effectively block the binding of SARS‐CoV‐2 to host cells. The cytotoxicity of nanoMIP was evaluated by MTT assay, the nanoMIP exhibited negligible cytotoxicity in HEK293T‐ACE2 cells even at concentrations up to 600 µg mL^−1^ (Figure S22a, Supporting Information), suggesting its superior cytocompatibility.

**Figure 4:**
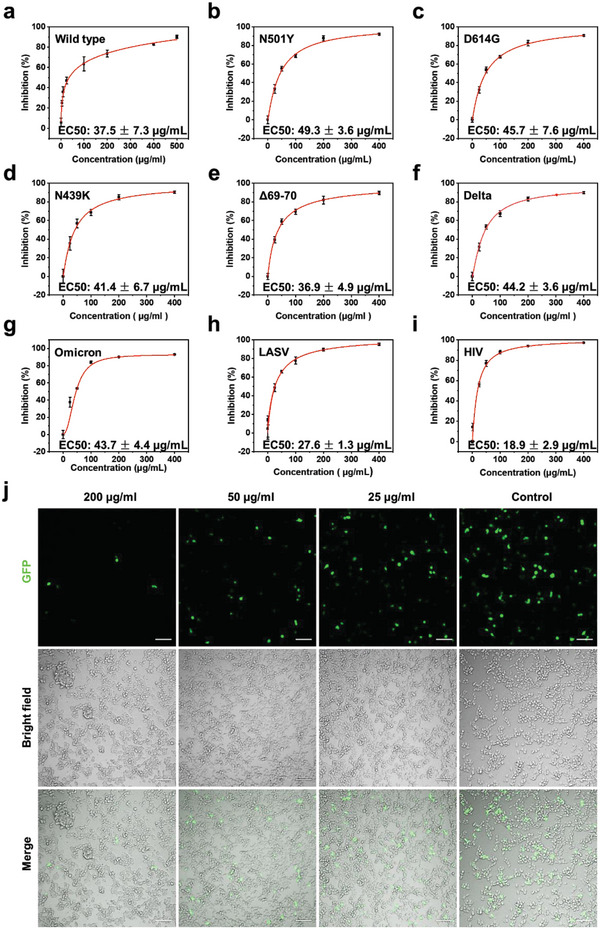
Broad‐spectrum antiviral capability of nanoMIP. a–i) Dose‐dependent inhibition of pseudovirus infectivity of nanoMIP. Wild type and mutant (N501Y, D614G, N439K, Δ69‐70, and variant of concern (VOC) Delta, Omicron) SARS‐CoV‐2 pseudovirus, LASV, and HIV‐1 pseudovirus were tested, respectively. The nanoMIP was incubated with pseudovirus for 30 min and the mixture was then added into wells containing ACE2‐HEK293T cells. Mean ± SD, *n*  =   3. j) Representative confocal fluorescence images of ACE2‐HEK293T cells without or with different concentrations of nanoMIP under the infection of SARS‐CoV‐2 pseudovirus (Omicron) for 48 h. Green represents the fluorescent proteins in the infected cells. Scale bar: 100 µm.

A pseudotyped virus, which was packed with the SARS‐CoV‐2 spike (wild type) and contained the genome of a green fluorescent protein (GFP) and a luciferase reporter, was employed for pseudovirus neutralization assay. As shown in **Figure** **4**a, determined by luciferase activity at 48 h after exposure to pseudovirus, the nanoMIP exhibited a maximum 90.2% inhibition efficiency to wild‐type pseudovirus of SARS‐CoV‐2, giving an EC50 value of 37.5 ± 7.3 µg mL^−1^. By contrast, much lower inhibition efficiency (≈20%) was observed for NIP (Figure S23a, Supporting Information) and nanoMIP saturated with RNase B protein (Figure S26, Supporting Information), which also implies that the inhibition efficiency of the nanoMIP was attributed to the binding toward high mannose. In addition, the expression of GFP after infection was also investigated. In the absence of the nanoMIP, host cells were susceptible to pseudovirus infection, showing intensive GFP expression after 48 h incubation. Comparatively, the GFP expression decreased with increasing the usage of nanoMIP (Figures S24 and S25, Supporting Information), which is consistent with luciferase reporting results.

In the last 2 years, several SARS‐CoV‐2 variants have exhibited higher transmissibility or severity compared to wild type and are less susceptible to some available vaccines or treatment regimens,^[^ ^46^ ^]^ which calls for new strategies capable to respond to different mutant strains. Considering most N‐glycosylation sites of S protein remain among typical SARS‐CoV‐2 variants^[^ ^7^, ^8^, ^9^ ^]^ and the content of high mannose in glycan shields changes slightly,^[^ ^20^, ^21^, ^47^, ^48^ ^]^ we speculated that high‐mannose binding nanoMIP should hold potential to protect host cells from variants infection. Hence, the inhibitory efficacy of nanoMIP against several typical mutant pseudoviruses (N501Y, D614G, N439K, and Δ69‐70) and multisite mutant pseudovirus (B.1.617.2, Delta) were investigated. As expected, the nanoMIP exhibited ≈90% neutralization efficacy for all tested pseudovirus variants, showing EC50 values of 49.3 ± 3.6 µg mL^−1^ for N501Y mutation (Figure 4b), 45.7 ± 7.6 µg mL^−1^ for D614G mutation (Figure 4c), 41.4 ± 6.7 µg mL^−1^ for N439K mutation (Figure 4d), and 36.9 ± 4.9 µg mL^−1^ for Δ69‐70 mutation (Figure 4e). As is widely reported, variant D614G favors an open conformational state, exhibiting efficient replication ex vivo and transmission in vivo,^[^ ^49^ ^]^ while variant N501Y is associated with increased receptor affinity.^[^ ^50^ ^]^ Intriguingly, the nanoMIP still showed a neutralizing effect on these strains similar to that of the wild‐type strain. In addition, as for the Delta variant, which once become a dominant strain worldwide^[^ ^8^ ^]^ with immune evasion due to antigenic drift, the nanoMIP bound on the changeless glycan shields and overcame virus diversity with an EC50 value of 44.2 ± 3.6 µg mL^−1^ (Figure 4f). In contrast, the NIP showed no obvious inhibition of the Delta variant (Figure S23b, Supporting Information). This result also indicated that the binding with high mannose is necessary but not sufficient for virus inhibition, which further demonstrated the superiority of nanoMIP as an antiviral agent. To be noted, the SARS‐CoV‐2 B.1.1.529 variant (Omicron) greatly reduced the neutralization potency of several treating NAb drugs (LY‐CoV016/LY‐CoV555, AZD1061/AZD8895, REGN10933/REGN10987, and BRII‐196).^[^ ^9^ ^]^ Therefore, we evaluated the inhibition efficacy of the nanoMIP against Omicron. In spite of the multiple mutations of the Omicron variant, the nanoMIP blocked nearly 90% of Omicron mutated pseudovirus from infecting cells with an EC50 value of 43.7 ± 4.4 µg mL^−1^ (Figure 4g,j). Our strategy shifts the focus from the causative viruses to the glycan shields and thus overcomes virus diversity. Together, the hypervalent anti‐high mannose nanoMIP exhibited broad‐spectrum and potent inhibition efficacy to SARS‐CoV‐2 major variants.

To further verify the potency of the broad‐spectrum antiviral strategy, the neutralization capability of nanoMIP to LASV and HIV‐1 pseudoviruses was also investigated. The cytotoxicity of the nanoMIP to host cells was studied and the nanoMIP was found to exhibit no obvious cytotoxicity to TZM‐bl cells (Figure S22a, Supporting Information). In the case of LASV pseudovirus, the nanoMIP blocked around 95.5% of pseudovirus from infecting cells, showing an EC50 value of 27.6 ± 1.3 µg mL^−1^ (Figure 4h). In addition, the nanoMIP exhibited 97.2% pseudovirus blocking against HIV‐1 pseudovirus, with an EC50 value of 18.9 ± 2.9 µg mL^−1^ (Figure 4i), while the NIP showed no obvious anti‐virus efficacy (Figure S23c, Supporting Information). The neutralization efficacy of LASV or HIV‐1 was higher than that of the SARS‐CoV‐2 pseudovirus. This could be attributed to the higher content of unprocessed oligomannose in glycan shields of LASV and HIV‐1.^[^ ^21^ ^]^ Taken together, the nanoMIP has been demonstrated to hold broad‐spectrum antiviral efficacy to multiple viruses including major SARS‐CoV‐2 variants and other vital viruses. Particularly, considering the apparent “molar mass” was estimated to be ≈36 000–50 000 kDa (see “Estimation of binding valency and apparent molecular mass of nanoMIP” in Experimental Section), the EC50 values for the viruses investigated were all around 10^−9^ m, which is comparable to that of many neutralizing antibodies.

### 2.4 Viral Aggregation Induced by NanoMIP

**Figure 5:**
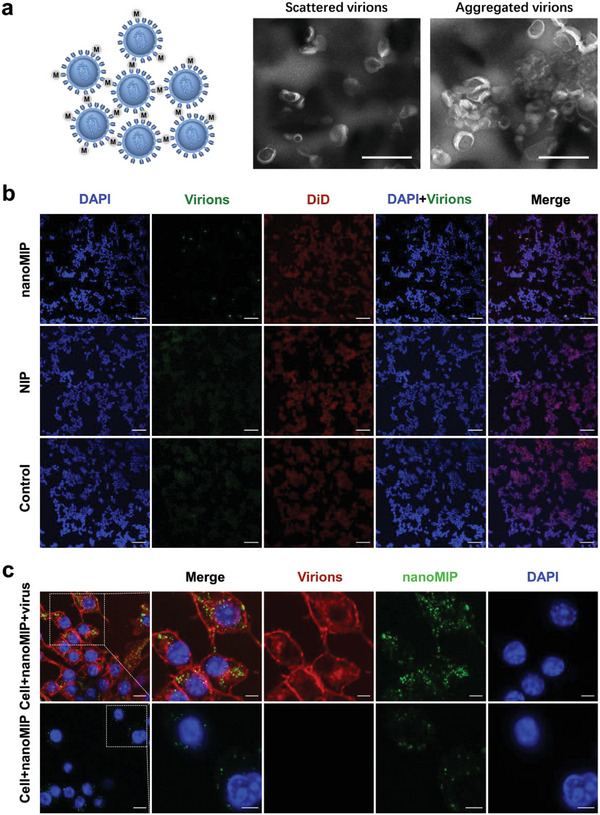
Viral aggregation induced by nanoMIP. a) Schematic of formation of virus aggregates induced by 200 µg mL^−1^ of nanoMIP (left). Negatively stained transmission electron microscopy images for scattered virions and aggregated virions induced by 200 µg mL^−1^ of nanoMIP (right). Scale bars, 500 nm. b) Representative confocal fluorescence images of SARS‐CoV‐2 pseudovirus treated with 200 µg mL^−1^ of nanoMIP, NIP, and nothing, respectively, in the presence of ACE2‐HEK293T cells. Red represents the cell membrane stained by DID, green represents the virions stained by DIO and blue represents the nucleus stained by DAPI Scale bars, 100 µm. c) Confocal fluorescence images for macrophage uptake of nanoMIP and virus aggregates induced by nanoMIP (the middle image without ruler represents the lack of corresponding channel information, 200 µg mL^−1^ of nanoMIP was used). Red represents the virions stained by DID, green represents the FITC‐doped nanoMIP, and blue represents the nucleus stained by DAPI. Scale bar: 10 µm (5 µm for enlarged images).

Encouraged by the potency of pseudovirus neutralization, the inhibition mechanism of glycan‐shield binding nanoMIP was further investigated. Negatively stained high‐resolution transmission electron microscopy (HR‐TEM) was employed to observe the morphology of wild‐type SARS‐CoV‐2 pseudovirus virions treated with or without the nanoMIP. For the control group, virions without treatment were scattered as individuals distinctly and easily observed. In contrast, virions treated with the nanoMIP were mostly in clusters, with few individual virions observed outside these clusters (**Figure** **5**a and Figures S19–S21, Supporting Information). The cross‐linking ability of the nanoMIP was further confirmed by confocal fluorescence microscopy. Fluorescence‐labeled SARS‐CoV‐2 pseudovirus (wild‐type) with different treatment were incubated with host cells. The fluorescence images showed that the nanoMIP could efficiently cross‐link virions and virions aggregated around the cell membrane without entry when compared with the group treated with the NIP and the control group (Figure 5b). Pseudovirus aggregates induced by the nanoMIP were of submicron to micron in size according to the time‐dependent virus aggregation test (Figure S33, Supporting Information), which would further contribute to blocking viral entry and reducing infection of host cells. Since the nanoMIP particles have been demonstrated to provide a large number of binding sites for high mannose in glycan shields of virions (Figure 1c,d), the cross‐linking ability of the nanoMIP should have originated from the simultaneous binding to multiple virion particles. Particularly, in the face of complex and changeable viruses, as long as they are modified with certain high mannose glycans in the glycan shields, it is possible to induce aggregation. This mechanism is general and can effectively suppress multiple viruses.

Next, the immune effect of virus aggregates induced by nanoMIP was further evaluated by macrophage phagocytosis. Macrophages, as a class of phagocytes, are responsible for engulfing and clearing pathogens, microbes, and other foreign intruders. For blank control, fluorescein isothiocyanate isomer (FITC)‐labeled nanoMIP (Figure S11, Supporting Information) alone was incubated with RAW 264.7 cells, confocal fluorescence images (Figure 5c) showed that few nanoMIP particles were taken up by macrophages, as is the same for pseudovirus without high‐mannose glycans group (Figure S36, Supporting Information). In contrast, significantly enhanced uptake of nanoMIP particles along with pseudovirus virions was observed when macrophages were incubated with the mixture of 4‐chlorobenzenesulfonate salt (DiD)‐labeled SARS‐CoV‐2 pseudovirus and FITC‐doped nanoMIP. As shown in Figure 5c and Figure S35, Supporting Information, the green FITC‐doped nanoMIP and the red DiD‐labeled pseudovirus were mostly co‐localized, demonstrating that pseudovirus and the nanoMIP were taken up as aggregates, totally different from DID‐only control (Figure S34, Supporting Information). Moreover, the viral gene expression of the pseudovirus after nanoMIP treatment and macrophage uptake was evaluated. Confocal fluorescence images (Figure S28, Supporting Information) showed that no GFP expression was observed, which proved that virus was inactivated. Since macrophages' phagocytosis is highly dependent on size, pseudovirus aggregates induced by the nanoMIP were at submicron to micron levels, which are exactly favorable for uptake by macrophages.^[^ ^51^, ^52^, ^53^ ^]^ Thus, we can conclude that the viral aggregation induced by the nanoMIP could well enhance macrophage phagocytosis, activate innate immunity, and facilitate the cleavage of the virus, which would further inhibit viral entry into host cells. Above all, this unique activating phagocytosis mechanism endowed high mannose‐binding nanoMIP with outstanding potential for COVID‐19 prevention and therapy.

### 2.5 Potent Broad‐Spectrum Inhibition of Authentic SARS‐CoV‐2

**Figure 6:**
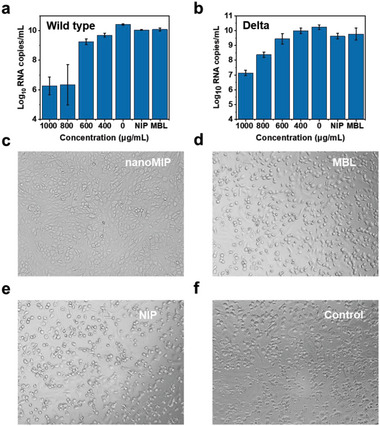
Potent inhibition of live viruses. a,b) The authentic SARS‐CoV‐2 virus (wild type and Delta) RNA load at 3 days post‐infection from Vero cells treated with different concentrations of nanoMIP. Mean ± SD, *n*   =   3. c–f) Cytopathic effect (CPE) images of Vero cells treated with nanoMIP (800 µg mL^−1^), NIP (800 µg mL^−1^), and MBL (10 µg mL^−1^) under the infection of live SARS‐CoV‐2 for 3 days.

Inspired by the potency in pseudovirus neutralization and viral aggregation induced by the nanoMIP, authentic virus neutralization was performed using both wild‐type and Delta SARS‐CoV‐2 viruses. MTT assay demonstrated the biosafety of the nanoMIP to Vero cells (Figure S22b, Supporting Information). As expected, the nanoMIP suppressed the infection of authentic SARS‐CoV‐2 to host cells successfully. Supernatants were harvested at 3 days post‐infection and tested utilizing real‐time quantitative polymerase chain reaction (RT‐qPCR) to quantify the copy number of viral genomes. As shown in **Figure** **6**a,b, with the increased dose of nanoMIP, the RNA load decreased for both wild‐type and Delta authentic SARS‐CoV‐2 viruses. Under the maximum dose, the viral RNA load dropped significantly, with about 3–4 orders of magnitude lower than the control group, for both wild‐type and Delta variants, which is comparable to neutralizing antibodies.^[^ ^54^ ^]^ Additionally, little‐to‐no cytopathic effect (CPE) was observed on the Vero cells monolayer treated with nanoMIP after infection for 72 h, while Vero cells treated with NIP or MBL showed obvious CPE (Figure 6c–f). Such an excellent antiviral effect was supposed to benefit from blocking the virus entry and inducing viral aggregation by the nanoMIP. Overall, these results demonstrated that the nanoMIP harbored potent broad‐spectrum antiviral efficacy due to its hypervalent binding to high‐mannose glycans of the glycan shields of viruses. As such, the high mannose‐binding nanoMIP holds outstanding potential for infectious disease prevention and therapy.

## 3 Conclusion

In this study, we developed a hypervalent glycan‐shield binding artificial antibody with nanoscale and rigid structure toward potent and broad‐spectrum inhibition of multiple viruses. Our controllably synthesized nanoMIP was endowed with the hypervalent binding capability to mannose (≈2576 binding sites on a single nanoparticle). Notably, the multivalently synergetic binding with high‐mannose glycans dramatically improved binding strength by 2–3 orders of magnitude compared with that toward mannose. When nanoMIP bonded with virions that are modified with a number of high‐mannose glycans, the binding strength was further enhanced by 2–3 orders of magnitude. Taking wild‐type SARS‐CoV‐2 pseudovirus as an example, the *K*_d_ value of nanoMIP reached 8.5 × 10^−10^ m. The high binding avidity, coupled with steric effect and rigid structure, made the nanoMIP capable of effectively blocking the interaction of virus and host cells. On the other hand, the nanoMIP exhibited additional unique strength that low‐valent neutralizing reagents fail to provide. Via simultaneous binding with multiple virions, the nanoMIP could induce viral aggregation, which could not only block the entry of virions into host cells but also facilitate the phagocytosis of virions. The nanoMIP exhibited potent inhibition toward pseudoviruses of the wild‐type strain, several major variants including D614G, N501Y, N439K, Δ69‐70, as well as the VOC Delta and Omicron strains. The EC50 values of the nanoMIP for the viruses investigated were all around 10^−9^ m, showing its high neutralization potency. Due to such a unique double‐punch (blocking and cross‐linking) mechanism, the hypervalent mannose‐binding nanoMIP provided an unprecedented strategy for potent and broad‐spectrum inhibition of different species of viruses, shifting the focus from the changeful and mutating peptide antigens or epitopes in conventional strategies (such as antibodies and vaccines) to the glycan shields, thus, overcoming the issues associated with virus diversity and mutation. We foresee the unique strength and great prospect of glycan‐shield binding nano‐artificial antibodies in the anti‐COVID‐19 fight, as well as broad‐spectrum inhibition toward other fatal viruses that would imperil public health emerging in the future.

## 4 Experimental Section

### Preparation of Man‐Imprinted Nanoparticles

The Man‐imprinted nanoparticles (nanoMIP) were synthesized according to the reverse microemulsion imprinting approach^[^ ^40^ ^]^ reported previously with major modifications. The procedure is shown in Figure 1a, which was composed of four steps as follows.

### Microemulsion Formation along with Template Anchoring

1.77 g of Triton X‐100, 100 mg amphiphilic template Man‐Bn, 7.5 mL of cyclohexane, 1.6 mL of n‐hexanol, 100 µL of ammonium hydroxide, and 480 µL of water were added to a 25‐mL eggplant flask with a 1.5 cm magnetic stir bar. The mixture was stirred at 700 rpm overnight at room temperature to form a clear and transparent solution.

### Reverse Microemulsion‐Confined Interface Imprinting

To the stabilized microemulsion system, 92 µL of TEOS was dropwise added carefully, then 8 µL of DFFPBA‐APTES (synthetic compound 4; 25 wt% in MeOH). The clear and transparent solution was kept stirring at 700 rpm at room temperature for 24 h. After that, 20 µL of TEOS/APTES (V:V, 5:3) was added slowly to the solution. The mixture was stirred at 700 rpm at room temperature for 12 h. For the preparation of FITC doped nanoMIP, 2 mg of FITC was added to the microemulsion in advance while the other procedures were the same as that for non‐doped nanoMIP.

### Microemulsion Disruption along with Template Removal

After imprinting, the prepared Man‐imprinted nanoparticles were released from the emulsion by adding 6 mL of acetone, then centrifuged for 30 min at 4000 rpm in order to separate the nanoMIP from the suspension. The obtained nanoparticles were washed along with ethanol and water successively three times, then nanoparticles were dispersed into 20 mL of 100 mm HAc (aq.) and shaken at room temperature for 20 min. The elution process shown above was repeated three times, and after removing the amphiphilic template Man‐Bn, the prepared nanoparticles were collected via centrifuging for 30 min at 4000 rpm. The nanoMIP was then washed along with PBS (1×), ethanol, and water successively, then lyophilized overnight and stored at 4 °C for future use.

### Surface PEGylation

170 µL of PBS (1×) and 10 mg of mPEG‐NHS were added to 1 mL solution of 10 mg mL^−1^ nanoparticles in water, the reaction mixture was stirred vigorously for 24 h at room temperature. The PEGylated Man‐imprinted nanoparticles were collected by centrifugation (8000 rpm, 20 min). The obtained nanoparticles were washed with anhydrous ethanol and water substantially, then lyophilized overnight and yielded as a white solid powder (15 mg per flask). The nanoMIP was dispersed into PBS (1×), sterilized by UV irradiation, and saved as 10 mg mL^−1^ stock solution at 4 °C for further use.

NIP was prepared using the same procedure except for the absence of amphiphilic template Man‐Bn.

### Estimation of Binding Valency and Apparent Molecular Mass of NanoMIP

To evaluate the binding capacity of Man‐imprinted nanoparticles, the number (*N*) of binding sites toward mannose and RNase B (with only one glycosylation site, high mannose type) on each nanoparticle was calculated as given below

(1) $$N=\frac{\frac{4}{3}\pi R^{3}\rho Q\max N_{A}}{M}$$

where *R* is the radius of a single nanoparticle (*R* ≈ 20 nm), *Q*_max_ is the saturated adsorption amount of mannose or RNase B bound by nanoMIP particles in terms of UV absorbance, *ρ* is the density of silica (*ρ* = 2.2 g cm^−3^), *N*_A_ = 6.02 × 10^23^ mol^−1^, *M*_RNase B_ = 14 700 Da, *M*_Man_ = 270 g mol^−1^.

The apparent molar mass of nanoMIP was estimated according to the equation below

(2) $$M_{\mathrm{nanoMIP}}=\frac{4}{3}\pi R^{3}\rho N_{A}$$

Considering the mean diameter of nanoMIP was 39.5 ± 2.3 nm (Figure S8, Supporting Information), the apparent molar mass of nanoMIP was estimated to be ≈36 000–50 000 kDa.

### Affinity and Competition Experiments via Biolayer Interferometry

The experiments were carried out on an Octet RED96 instrument (Molecular Devices, ForteBio). All *K*_d_ values were calculated with the use of a 1:1 global fit model. For affinity determination of nanoMIP with RNase B, SARS‐CoV‐2 S1, and HIV‐1 GP120, nanoMIP was loaded at 100 µg mL^−1^ in kinetics buffer (PBS, 1×) for 5 min onto aminopropylsilane (APS) biosensors. Association of RNase B, SARS‐CoV‐2 S1, and HIV‐1 GP120 was performed in kinetics buffer solution (0.2‰ Tween‐20 in PBS, 1×) at 250, 500,1000, 1500, and 2000 nm for 300 s, respectively. The dissociation process in kinetics buffer solution (0.2‰ Tween‐20 in PBS, 1×) was measured for 300 s. For affinity determination of NIP with RNase B, SARS‐CoV‐2 S1, and HIV‐1 GP120, all the procedures were the same as described above except that the nanoMIP used was changed to the NIP.

Alternatively, apparent *K*_d_ for nanoMIP was measured using Ni‐NTA biosensors. RNase B and SARS‐CoV‐2 S1 were loaded for 300 s at 100 nm in kinetics buffer solution (0.2‰ Tween‐20 in PBS, 1×) onto Ni‐NTA biosensors. Curves for association were recorded for 300 s via incubating the protein‐coated sensors with several concentrations of nanoMIP. Curves for dissociation were recorded for another 300 s by moving the sensors to buffer wells (0.2‰ Tween‐20 in PBS, 1×).

For the affinity determination of nanoMIP with SARS‐CoV‐2 pseudovirus (wild type), the original solution of pseudovirus was diluted by 100 times and loaded in kinetic buffer (PBS, 1×) for 5 min onto APS biosensors. Association of nanoMIP with different concentrations was performed in kinetics buffer solution (0.2‰ Tween‐20 in PBS, 1×) for 5 min. Dissociation in kinetics buffer solution (0.2‰ Tween‐20 in PBS, 1×) was measured for 5 min. For negative controls, pseudovirus was digested by PNGase F enzyme, or the nanoMIP was pre‐treated with 10 mg mL^−1^ RNase B solution, while other procedures were the same as above. For ACE2 competition experiments at the protein level, nanoMIP was loaded at 100 µg mL^−1^ in kinetics buffer (PBS, 1×) for 5 min onto APS biosensors. Association curves of SARS‐CoV‐2 S1 with different concentrations in kinetics buffer solution (0.2‰ Tween‐20 in PBS, 1×) were recorded for 5 min. Then, the association of ACE2 was performed in kinetics buffer solution (0.2‰ Tween‐20 in PBS, 1×) at 100 nm for 300 s. Curves for dissociation in kinetics buffer solution (0.2‰ Tween‐20 in PBS, 1×) was measured for 5 min. For ACE2 competition experiments at the pseudovirus level, the original solution of pseudovirus was diluted by 100 times and loaded in kinetic buffer (PBS, 1×) for 5 min onto APS biosensors. Association curves of nanoMIP with different concentrations in kinetics buffer solution (0.2‰ Tween‐20 in PBS, 1×) were recorded for 5 min, and then, the association of ACE2 was performed in kinetics buffer solution (0.2‰ Tween‐20 in PBS, 1×) at 100 nm for 300 s. Curves for dissociation in kinetics buffer solution (0.2‰ Tween‐20 in PBS, 1×) was measured for 5 min.

### Pseudovirus Attachment and Blocking

To study the blocking effect of nanoMIP on virus attachment, the virions of SARS‐CoV‐2 pseudovirus were labeled with 3,3‐dioctadecyloxacarbocyanine perchlorate (DiO).^[^ ^55^ ^]^ Briefly, 200 µL of pseudovirus stock solution (wild type) was incubated with 10 µL of 20 µm DiO (ethanol) for 30 min in dark, then centrifuged at 4000 rpm for 30 min at 4 °C and rinsed with 100 µL of phosphate buffer (10 mm, pH 7.4) twice to remove the residual dye. Afterward, 100 µL of DiO‐labeled virion was incubated with 900 µL of 1 mg mL^−1^ nanoMIP suspension in PBS for 30 min on a shaking table at 300 rpm. The mixture was then applied to HEK293T‐ACE2 cells, which were adhered to the culture dish, and incubated at 4 °C for 1 h. Unbound virus particles were then removed by washing extensively with PBS (1×) three times. Afterward, the cells were stained by 4,6‐diamidino‐2‐phenylindole (DAPI) for 10 min, then visualized by confocal fluorescence microscopy (Zeiss LSM 710, Germany). For the NIP group, all the procedures were the same as described above except that the nanoMIP was changed to NIP, and for the control group, the nanoMIP was absent and other procedures were the same. For DIO‐only control, both the pseudovirus and nanoMIP were absent, in which Leica SP8 was used. For negative control, the pseudovirus was replaced by that digested via the PNGase F enzyme. The flow cytometry was performed with a similar procedure on the Beckman Coulter CytoFLEX S system (California, USA) with more than 10 000 cells being analyzed.

### Pseudovirus Neutralization Assays

For the SARS‐CoV‐2 spike pseudotyped HIV‐1 neutralizing assays (wild type, D614G, N501Y, N439K, Δ69‐70, and Omicron), HEK293T‐ACE2 cells were seeded in a 96‐well microplate at the density of 1 × 10^4^/well overnight. Different concentrations of nanoMIP suspension (25, 50, 100, 200, and 400 µg mL^−1^) in DMEM culture medium were incubated with 20 000 TU of SARS‐CoV‐2 spike protein pseudotyped HIV‐1 for 30 min at 37 °C. The virus‐MIP solution was added into wells containing HEK293T‐ACE2 cells and incubated for 48 h (*n* = 3 for each group, 37 °C, 5% CO_2_). After the incubation, the cells were imaged with a fluorescence microscope. Besides, the emissions of GFP were also captured by confocal fluorescence microscopy (Zeiss LSM 710, Germany) after DAPI treatment. Furthermore, the treated cells were lysed, and then, the luciferase activity was determined by means of a firefly luciferase assay kit (Yeasen, China). The luminescence intensity of each well was measured by a microplate reader (Winooski, VT, USA), and the neutralization rate was calculated as the equation

(3) $$\begin{array}{c} \begin{array}{ccc} \mathrm{Neutralization}\;\mathrm{rate} & = & \left(1-\frac{\mathrm{Intensity}\;(\mathrm{test}\;\mathrm{sample})-\mathrm{Intensity}\;(\mathrm{blank}\;\mathrm{control})}{\mathrm{Intensity}\;(\mathrm{virus}\;\mathrm{control})-\mathrm{Intensity}\;(\mathrm{blank}\;\mathrm{control})}\right)\\ & & \times \;100\% \end{array} \end{array}$$

where blank control is the group cells without virus and nanoMIP, and virus control is the group cells treated only with the virus. Three parallel samples were conducted for each group and the half‐maximal effect concentration (EC50) value was fitted.

For the SARS‐CoV‐2 spike pseudotyped VSV (Delta)neutralizing assay, 1000 TCID_50_ SARS‐CoV‐2 spike pseudotyped VSV (delta) was added to each well. Moreover, for the LASV pseudovirus neutralizing assay, 1000 TCID_50_ LASV pseudovirus was added to each well and HEK293T cells were employed instead of the HEK293T‐ACE2 cells, and for the HIV‐1 pseudovirus neutralizing assay, 200 TCID_50_ HIV‐1 pseudovirus with 15 µg mL^−1^ DEAE pro‐infective agent was added to each well and the HEK293T‐ACE2 cells were changed to the TZM‐bl cells. All the procedures were the same as described above except for the GFP observation. For the NIP group, all the procedures were the same as described above except that the nanoMIP was changed to NIP. For negative control, the nanoMIP was pre‐treated with 10 mg mL^−1^ RNase B solution, and other procedures were the same as above.

### Authentic Virus Neutralization Assay

Vero cells were seeded in a 96‐well microplate overnight. The nanoMIP was serially diluted in DMEM culture medium and mixed with 10 TCID_50_ live SARS‐CoV‐2 viruses for 30 min at 37 °C. The virus‐MIP solution was added into wells containing Vero cells and then incubated for 1 h (*n* = 3 for each group, 37 °C, 5% CO_2_). Subsequently, cells were overlaid with different concentrations of nanoMIP in DMEM supplemented with 2% FBS and incubated for 3 days (37 °C, 5% CO_2_). The CPE was examined for 3 days post‐infection and imaged by a microscope. Supernatants were harvested at 3 days post‐infection and tested utilizing real‐time quantitative PCR to quantify the number of viral genomes. Amplification was performed utilizing a One Step PrimeScript RT‐PCR Kit (RR064A, TaKaRa). Cells without nanoMIP were used as virus controls. For NIP or MBL group, all the procedures were performed in a similar way as described above except that the nanoMIP was changed to NIP or MBL.

### Cryo‐Transmission Electron Microscopy

For binding experiments, the nanoMIP (25 µg mL^−1^) was incubated with SARS‐CoV‐2 pseudovirus (wild type) for 30 min. Then, 5 µL of the sample solution was pipetted to the hydrophilized microscopical 200 mesh grid (R1/4 batch of Quantifoil, MicroTools GmbH, Jena, Germany) and the supernatant was immediately removed with a piece of filter paper. As a result, an ultrathin layer of the sample solution was obtained by spanning the holes of the carbon film. The sample was instantly vitrified by plunging the grid into liquid ethane and subsequently transferred under liquid nitrogen into a Talos F200C TEM (FEI Company, OR, USA) and operating at 200 kV by the use of a Gatan cryo‐holder (Model 626). Micrographs were taken with a 4k × 4K Ceta CMOS camera at binning 1 mode, and for pseudovirus Cryo‐TEM experiments, nanoMIP was absent in the test samples.

### Negative‐Stain Transmission Electron Microscopy

To evaluate the binding effect of nanoMIP on viral particles, SARS‐CoV‐2 pseudovirus (wild type) was pretreated with 200 µg mL^−1^ nanoMIP suspension for 1 h. Then the mixture was pipetted to a copper mesh and settled for 1 min. The floating liquid was absorbed by filter paper. 3% phosphotungstic acid was added onto the copper mesh for 1 min, then filter paper was utilized as described above. After removing the solution, the grid was air‐dried at room temperature for 24 h and imaged with a JEM‐2100F TEM (JEOL, Tokyo, Japan) at 200 kV. For control experiments, nanoMIP was absent in the test samples.

### Statistical Analysis

Data are shown as mean ± SD, *n*  =  3 in all cases, and all the biolayer interferometry *K*_d_ values were calculated by DataAnalysisHT12 software.

## Conflict of Interest

The authors declare no conflict of interest.

## Supporting information

Supporting Information

## Data Availability

The data that support the findings of this study are available from the corresponding author upon reasonable request.
