# Supplementary material for: Rational Development of Hypervalent Glycan Shield‐Binding Nanoparticles with Broad‐Spectrum Inhibition against Fatal Viruses Including SARS‐CoV‐2 Variants
Source: Adv Sci (Weinh). 2022 Nov 15;10(2):2202689. doi: 10.1002/advs.202202689 (PMC9839850; doi:10.1002/advs.202202689)
Supplement: Supplementary file 1 — Supporting Information [file ADVS-10-2202689-s001.pdf]

Supporting Information

Rational development of hypervalent glycan shield-binding nanoparticles with broad-spectrum inhibition against fatal viruses including SARS-CoV-2 variants

Ying Li, Shuxin Xu, Qing Ye, Hang Chi, Zhanchen Guo, Jingran Chen, Mei Wu, Baochao Fan, Bin Li, Cheng-Feng Qin and Zhen Liu**

MATERIALS AND METHODS

Reagents and materials

Transferrin (TRF), horseradish peroxidase (HRP), bovine albumin (BSA), catalase and Triton X-100 were obtained from Sigma-Aldrich (St. Louis, MO, USA). His-tagged SARS-CoV-2 S1 protein (S1N-C52H3), His-tagged human ACE2 (AC2-H52H8), His-tagged HIV GP120 (HIV-1 CN54) and mannose-binding lectin (MBL, MBL-H5220) were purchased from ACROBiosystems (Beijing, China). D-(+)-glucose, L-(-)-fucose, D-(+)-galactose, N-acetyl-D-glucosamine, and 2,4-difluoro-3-formylphenylboronic acid were purchased from Bide Pharmatech (Shanghai, China). D-(+)-mannose, ribonuclease B (RNase B), benzyl alcohol, anhydrous sodium sulphate, sodium bicarbonate, sodium chloride, cyclohexane, sodium methoxide, anhydrous iron(III) chloride, dichloromethane, dry 1,2-dichloroethane, ethyl acetate, 4A molecular sieve and N-hydroxylsuccinimide-polyethylene glycol (mPEG-NHS) (MW: 350 Da, 750Da, 1000Da, 2000Da, 5000Da) were obtained from Aladdin Reagent (Shanghai, China). Amberlite IR 120 (H+) resin was purchased from Alfa-Aesar (Shanghai, China. Aminopropyltriethoxysilane (APTES), tetraethyl orthosilicate (TEOS) and fluorescein isothiocyanate isomer (FITC) were purchased from J&K Scientific (Shanghai, China). 3,3-Dioctadecyloxacarbocyanine perchlorate (DiO), 4-chlorobenzenesulfonate salt (DiD), 1,1-dioctadecyl-3,3,3,3-tetramethylindodicarbocyanine, 4,6-diamidino-2-phenylindole (DAPI) and MTT assay kit were purchased from Beyotime Biotechnology (Shanghai, China). Acetic anhydride, anhydrous pyridine, ammonium hydroxide and anhydrous ethanol were purchased from Nanjing Reagent Company (Nanjing, China). Acetic acid (HAc) was purchased from Sinopharm Chemical Reagent Co. (Shanghai, China). Phosphate-buffered saline for cell culture (1× PBS), parenzyme cell digestion solution (containing 0.25% trypase and 0.02% EDTA), Dulbecco Modified Eagle Medium (DMEM, containing 4.5 mg/ml glucose, 80 U/ml penicillin and 0.08 mg/ml streptomycin), dimethyl sulfoxide (DMSO) and 96-well plates were purchased from Nanjing KeyGen Biotech (Nanjing, China). Fetal bovine serum (FBS) was purchased from Gibco (Life Technologies, Australia). Luciferase Reporter Gene Assay Kit was bought from Yeasen Biotechnology (Shanghai, China). Water used in all the experiments was purified by a Milli-Q Advantage A10 water purification system (Millipore, Milford, MA, USA). All chemicals were used directly without any further purification unless otherwise stated.

Instruments

Transmission electron microscopic (TEM) characterization was carried out on a JEM-2800 system (JEOL, Tokyo, Japan). Negative-stain Transmission Electron Microscopy was carried out on a JEM-2100F field-emission high-resolution transmission electron microscope (JEOL, Tokyo, Japan). Cryo-Transmission Electron Microscopy (Cryo-TEM) was carried out on a Talos F200C TEM (FEI Company, OR, USA) by the use of a Gatan cryo-holder (Model 626). Scanning electron microscope (SEM) characterization was carried out on a JSM-7800F system (JEOL, Tokyo, Japan). X-ray photoelectron spectroscopy (XPS) characterization was carried out on a K-Alpha X-ray

photoelectron spectrometer system (Thermo Fisher, MA, USA). Ultraviolent (UV) spectral analysis was performed with a NanoDrop 2000/2000C spectrophotometer (Thermo Fisher, MA, USA). Microplate reader analysis was carried out on a BioTek Synergy Mx microplate reader (Winooski, VT, USA). Bio-layer interferometry (BLI) binding assays were determined on an Octet Red 96 instrument (FroteBio, CA, USA) using aminopropylsilane (APS) or Ni-NTA biosensors. Excitation-emission spectra was carried on an FLS980 fluorescence spectrometer (Edinburgh Instruments, UK). Cell imaging was carried out on an LSM 710 laser scanning confocal microscopy (Zeiss, Oberkochen, Germany) or Leica SP8 laser scanning confocal microscopy (Leica, Wetzlar, Germany). Flow cytometric analysis was performed on a Beckman Coulter CytoFlex S system (California, USA). Fourier transform infrared (FT-IR) spectrometry was carried out on a Nicolet 6700 FT-IR spectrometer (Thermo Fisher, MA, USA). ^{1}H NMR (500 MHz), ^{13}C NMR (125 MHz) and ^{19}F NMR spectra were collected with a Bruker DRX 500 NMR spectrometer at room temperature (25 °C). Mass spectrum was collected on a OrbiTrap high resolution mass spectrometry (Thermofisher LTQ-Orbitrap XL, MA, USA). PCR amplification was conducted in a LightCycler® 480 Instrument (Roche Diagnostics Ltd, Rotkreuz, Switzerland).

Cells, pseudoviruses and authentic viruses

ACE2-transfected HEK293T cells (FBC2591), HEK293T cells, SARS-CoV-2 S pseudotyped HIV-1 (FNV215), SARS-CoV-2 S with N501Y mutation pseudotyped HIV-1 (FNV3247), SARS-CoV-2 S with D614G mutation pseudotyped HIV-1 (FNV2776), SARS-CoV-2 S with N439K mutation pseudotyped HIV-1 (FNV3226), SARS-CoV-2 S with Δ69-70 mutation pseudotyped HIV-1 (FNV3251) and SARS-CoV-2 S (B.1.1.529, Omicron) pseudotyped HIV-1 (FNV4122) were obtained from Fubio Biotechnology Co., Ltd. (Suzhou, China). SARS-CoV-2 S pseudotyped HIV-1 was packaged with spike protein of SARS-CoV-2 as the surface capsid glycoprotein and the RNA genome with the gene of GFP and luciferase. TZM-bl cells were purchased from Goybio (Shanghai, China). Vero cells and RAW264.7 cells were purchased from Nanjing KeyGen Biotech (Nanjing, China). SARS-CoV-2 S (B.1.617.2, delta) pseudotyped VSV (80048), HIV-192-36 pseudovirus (80004) and LASV pseudovirus (80034) were purchased from Tiantan Pharmaceutical Biotech (Beijing, China). All the experiments involving pseudoviruses were performed in a BSL-2 laboratory.

The SARS-CoV-2 strain BetaCoV/Beijing/IME-BJ01/2020 (No. GWHACAX01000000) was originally isolated from a COVID-19 patient in China early 2020. The SARS-CoV-2 Delta variant (CSTR.16698.06.NPRC 6.CCPM-B-V-049-2105-6) was isolated from an imported patient from India. All live SARS-CoV-2 virus infections were performed in a BSL-3 laboratory.

HEK293T-ACE2, HEK293T, Raw264.7, Vero or TZM-bl cells were cultured in DMEM medium with 10% fetal bovine serum (37 °C, 5% CO_2).

The PCR was conducted in a LightCycler® 480 Instrument (Roche Diagnostics Ltd, Rotkreuz, Switzerland). SARS-CoV-2 RNA was measured with the following primer-probe set: E_CoV2_F (5’-ACAGGTACGTTAATAGTTAATAGCGT-3’),

E_CoV2_R(5'-ATATTGCAGCAGTACGCACACA-3'), E_CoV2_P (5'-ACACTAGCCATCCTTACTGCGCTTCG-3').

Synthesis of amphiphilic template Man-Bn

To prepare Man-imprinted nanoparticles by microemulsion system, the amphiphilic template Man-Bn is necessary. Therefore, we synthesized Man-Bn according to approaches reported previously with major modifications. The synthetic route is composed of three steps (Figure S1): 1) Acetylation of mannose[1]; 2) Coupling of benzyl group[2]; 3) Deacetylation of mannoside[3], which is described in details as follows.

1,2,3,4,6-Penta-O-acetyl-α-D-mannopyranose

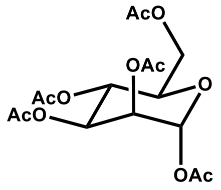

1

To a solution of D-(+)-mannose (3.06 g; 17.0 mmol; 1 equiv.) in dry pyridine (15 mL; 186 mmol;11 equiv.) at 0 °C under N_2 was added acetic anhydride (16 mL; 170 mmol; 10 equiv.). The reaction mixture was sealed and kept at room temperature for 24 h. The reaction was slowly poured into ice water (100 mL) and then extracted with dichloromethane (3 x 50 mL). The organic layer was washed with 1 M HCl (aq.) and saturated $NaHCO_3$ sequentially until the evolution of gases ceased, and then washed with water and brine successively. The organic phase was dried over Na_2SO_4 and condensed to yield 6.46 g of **1** (97.3%) as a sticky white solid.

2-Benzyl-2,3,4,6-tetra-O-acetyl-α-D-mannopyranoside

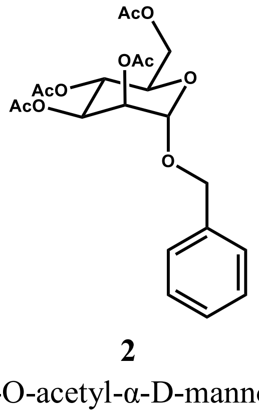

2

To a solution of 1,2,3,4,6-penta-O-acetyl-α-D-mannopyranose **1** (1.56 g; 4 mmol; 1 equiv.), anhydrous sodium sulfate (2 g) and anhydrous ferric chloride (0.649 g; 4 mmol; 1 equiv.) in dry 1,2-dichloroethane (40 mL) under N_2 atmosphere was added benzyl alcohol (0.692 g; 6.4 mmol; 1.6 equiv.). The reaction mixture was sealed and stirred vigorously at room temperature, while monitoring the reaction progress by TLC. Upon completion (8 h), the reaction was diluted with dichloromethane and neutralized with saturated $NaHCO_3$. The organic layer was then washed with $NaHCO_3$, H_2O and brine

successively. It was dried over $NaSO_4$, filtered and concentrated under reduced pressure. The crude product was purified by flash column chromatography to give mannoside **2** as yellow oil (1.40 g, 90.5%).

2-Benzyl-α-D-mannopyranoside (Man-Bn)

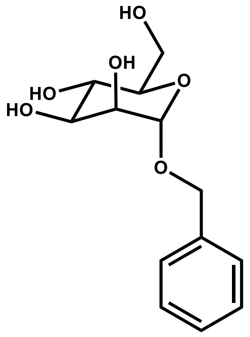

3

To a solution of 2-benzyl-2,3,4,6-tetra-O-acetyl-α-D-mannopyranoside **2** (11 g; 25 mmol; 1 equiv.) in dry MeOH (200 mL) under N_2 atmosphere was added sodium methoxide (5.4 g; 100 mmol; 4 equiv.). The reaction was then stirred at room temperature under N_2, monitoring by TLC. Upon completion (6 h), the mixture was neutralized with Amberlite IR 120 (H^+) resin. The resin was filtered and solvent was evaporated to give **3** as a hygroscopic white solid (6.1 g; 90.4%).

Synthesis of functional monomer DFFPBA-APTES

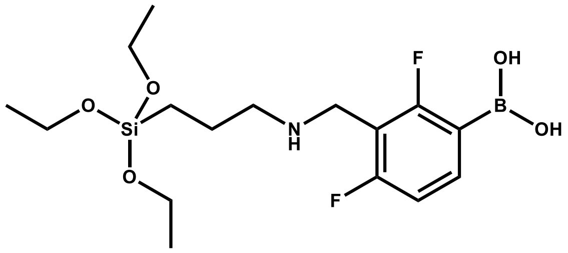

DFFPBA-APTES

The 3-aminopropyltriethoxysilane (5 ml; 20 mmol; 2 equiv.) and 2,4-difluoro-3-formylphenylboronic acid (1.86 g; 10mmol; 1 equiv.) were dissolved in dry MeOH (60 mL) with 4A molecular sieve (4 g) and the resulting solution was stirred under N_2 atmosphere at room temperature overnight, then sodium cyanoborohydride (1.26 g; 20 mmol; 2 equiv.) in anhydrous MeOH (20 mL) was added slowly. After 12 h, the solution was filtered and solvent was evaporated under reduced pressure. The crude product was washed with ethyl acetate and cyclohexane three times to afford DFFPBA-APTES as a white powder (3.22 g; 82.4%).

Optimization of the imprinting conditions for Man-imprinted nanoparticles

The specific monomer ratio of DFFPBA-APTES and TEOS used for the preparation of Man-imprinted nanoparticles were optimized in terms of the obtained imprinting factor (IF). Man-imprinted nanoparticles (2.0 mg each) prepared at different molar ratios of

monomers (DFFPBA-APTES and TEOS) were dispersed separately into 200 μL of phosphate buffer (10 mM, pH 7.4) containing 1 mg/mL of Man-Bn. After incubation at room temperature for 2 h, the nanoparticles were collected by centrifugation at 4,000 rpm for 30 min and rinsed with 200 μL of phosphate buffer (10 mM, pH 7.4) three times. The nanoparticles were re-suspended and eluted in 20 μL of 100 mM HAc (aq.) at room temperature for 1 h on a rotator. Finally, the suspensions were centrifuged at 4,000 rpm for 30 min and the supernatant were collected. The amount of Man-Bn was determined by measuring the UV absorbance of the supernatant at 230 nm. Three parallel samples were measured for each group. For control experiments, all the procedures were the same as described above except the absence of Man-Bn in the test samples. IF was calculated according the following equation:

$$\mathrm{IF}=\frac{\mathrm{Abs(\ Man\text{-}imprinted\ nanoparticles)\text{-}\ Abs(Control)}}{\mathrm{Abs(NIP)\text{-}Abs(control)}} \qquad (1)$$

Optimization of the PEG chain length of Man-imprinted nanoparticles

The PEG chain length used for the surface PEGylation of Man-imprinted nanoparticles was optimized in terms of the obtained imprinting factor (IF). Man-imprinted nanoparticles (2.0 mg each) coupled with different lengths of mPEG-NHS (MW: 350 Da, 750Da, 1000Da, 2000Da, 5000Da) were dispersed separately into 200 μL of phosphate buffer (10 mM, pH 7.4) containing 1 mg/mL of Man-Bn. After incubation at room temperature for 2 h, the nanoparticles were collected by centrifugation at 8000 rpm for 20 min and rinsed with 200 μL of phosphate buffer (10 mM, pH 7.4) three times. The nanoparticles were re-suspended and eluted in 20 μL of 100 mM HAc (aq.) at room temperature for 1 h on a rotator. Finally, the suspensions were centrifuged at 8000 rpm for 20 min and the supernatant were collected. The amount of Man-Bn was determined by measuring the UV absorbance of the supernatant at 230 nm. Three parallel samples were measured for each group. For control experiments, all the procedures were the same as described above except the absence of Man-Bn in the test samples. IF was calculated as described above.

Selectivity test of nanoMIP

The selectivity of PEGylated Man-imprinted nanoMIP at the monosaccharide level was evaluated using D-(+)-mannose, D-(+)-glucose, L-(-)-fucose, D-(+)-galactose and N-acetyl-D-glucosamine. To facilitate detection, monosaccharides were pre-modified with benzyl group. Firstly, each monosaccharide standard solution (1mg/mL) was separately prepared with phosphate buffer (10 mM, pH 7.4). Then equivalent nanoMIP and NIP (2 mg each) were added to 200 μL of the monosaccharide solutions in 250-μL microcentrifugal tubes. The tubes were shaken on a rotator at room temperature for 2 h. The nanoparticles were collected by centrifugation at 8000 rpm for 20 min and rinsed with 200 μL phosphate buffer (10 mM, pH 7.4) three times. Secondly, the nanoparticles were re-suspended and eluted in 20 μL of 100 mM HAc (aq.) at room temperature for 1 h on a rotator. Finally, the nanoparticles were precipitated via centrifugation and the supernatant were collected. The amount of monosaccharide bound by the nanoMIP was determined by measuring the UV absorbance of the supernatant at 230 nm. The

measurement was repeated three times. For control experiments, all the procedures were the same as described above except the absence of monosaccharide in the test samples.

For the selectivity test of PEGylated Man-imprinted nanoMIP at the protein level, all the procedures were the same as described above except that the test monosaccharides used were changed to the proteins including RNase B, TRF, HRP, BSA and catalase.

For the deposition experiment, 1 mL of 1 mg/mL nanoMIP suspension in PBS was applied to HEK293T-ACE2 cells, which were adhered to the culture dish, and incubated at 4 °C for 1 h. The cells were then visualized by the Zeiss LSM 710 or Leica SP8 confocal laser scanning fluorescence microscopy.

Measurement of adsorption isotherm

A series of standard solutions composed of Man-Bn with known concentrations were prepared with phosphate buffer (10 mM, pH 7.4). Firstly, 2 mg of the Man-imprinted nanoparticles were separately added to 200 μL of different concentrations of Man-Bn solution in 250-μL plastic microcentrifugal tubes, respectively. The tubes were shaken on a rotator for 2 h at room temperature. The nanoparticles were collected by centrifugation at 8000 rpm for 20 min and rinsed with 200 μL of phosphate buffer (10 mM, pH 7.4) three times. The nanoparticles were re-suspended and eluted in 20 μL 100 mM HAc (aq.) at room temperature for 1 h on a rotator. Finally, the suspensions were centrifuged at 8000 rpm for 20 min and the supernatant were collected. The amount of Man-Bn was determined by measure the UV absorbance of the supernatant at 230 nm. The measurement was repeated three times. An adsorption isotherm was established by plotting the UV absorbance at the 230 nm of the supernatant against the logarithmic concentration of Man-Bn. To estimate the binding affinity of Man-imprinted nanoparticles, the amount of Man-Bn bound by the Man-imprinted nanoparticles was plotted according to the Scatchard equation as given below:

$$\frac{Q_e}{[s]} = \frac{Q_{max}}{K_d} - \frac{Q_e}{K_d} \quad (2)$$

where Q_e, [s], Q_{max} and K_d are the amount of Man-Bn bound by the Man-imprinted nanoparticles in terms of UV absorbance at equilibrium, the free concentration at adsorption equilibrium, the saturated adsorption amount in terms of UV absorbance and the dissociation constant, respectively. By plotting Q_e/[s] versus Q_e, Q_{max} and K_d could be calculated from the slope and intercept, respectively.

For the measurement of adsorption isotherm at the protein level, all the procedures were the same as described above except that Man-Bn was replaced by RNase B and the UV absorbance was measured at 214 nm.

Cytotoxicity of nanoMIP *in vitro*

Cell viability was determined by the MTT assay. Briefly, HEK293T-ACE2, Vero or TZM-bl cells were seeded on 96-well microplates with a density around 1×10^4 cells per well and allowed to adhere overnight prior to the assay. The cells were then incubated with different concentrations of nanoMIP at 37 °C for 24 h, which were set as test groups. The cells without the treatment of nanoMIP were set as control group, wells

without cells were used as background group. After the incubation, 50 μL of MTT indicator dye (1 mg/mL in PBS) was added. After incubating for another 4 h at 37 °C in the dark, the supernatant was discarded, and 150 μL of DMSO was added to each well. After shaking for 10 min on a shaking table, the optical density of the solution was monitored on the microplate reader, and absorbance was measured at a wavelength of 550 nm. The cell viability was calculated by the following equation:

$$\text{Cell viability (\%)}=\frac{\text{Abs (test)-Abs (background)}}{\text{Abs (control)-Abs (background)}}\times 100\% \tag{3}$$

Pseudovirus aggregation induced by nanoMIP

To study virus aggregates induced by nanoMIP, 10 μL of DiO labelled virion were incubated with 90 μL of 200 μg/mL nanoMIP suspension in DMEM culture medium for 2 h at 300 rpm on a shaking table. The mixture was then applied to HEK293T-ACE2 cells, which were cultured in a 96-well microplate, and incubated for 1 h at 37 °C. Afterwards, the cell membrane was stained by 1,1-dioctadecyl-3,3,3,3-tetramethylindodicarbocyanine, 4-chlorobenzenesulfonate salt (DiD) and cell nucleus was stained with DAPI and visualized by confocal laser scanning microscopy (Zeiss LSM 710, Germany). For NIP group, all the procedures were the same as described above except that the nanoMIP was changed to NIP. And for control group, all the procedures were the same as described above except the absence of nanoMIP. For DIO-only control, both pseudovirus and nanoMIP were absent, for the imaging of which Leica SP8 was used. The time-dependent virus aggregation was observed at 0, 0.5, 1 and 2 h, respectively.

Membrane fusion assay

To study the effect of nanoMIP treatment on membrane fusion, HEK293T-ACE2 cells were seeded in a 96-well microplate and cultured overnight. Then the cell culture medium was discarded and washed once with PBS (1 x). After that, 50 μL of SARS-CoV-2 pseudovirus stock solution, 90 μL of 100 μg/mL nanoMIP suspension in DMEM culture medium were added the well. The cells were then cultured for another 24 h (37 °C, 5% CO_2) and washed extensively with PBS (1 x), stained with DAPI and visualized by confocal laser scanning microscopy (Zeiss LSM 710, Germany). Six replicate wells were set for this experiment. For control group, all the procedures were the same as described above except the absence of nanoMIP.

Macrophage uptake

To study macrophage (RAW264.7 cells) uptake of nanoMIP and pseudovirus aggregates via confocal microscopy, FITC-doped nanoMIP were prepared and SARS-CoV-2 pseudovirus (wild type) were labelled with DiD. Briefly, 200 μL of pseudovirus stock solution (wild type) was incubated with 10 μL of 20 μM DiD (ethanol) for 30 min in dark, then centrifuged at 4,000 rpm for 30 min at 4 °C and rinsed with 100 μL of phosphate buffer (10 mM, pH 7.4) twice to remove the residual dye. Afterwards, 100 μL of DiD labelled virion was incubated with 900 μL of 200 μg/mL FITC-doped nanoMIP suspension in DMEM culture medium for 30 min on a shaking table at 300

rpm. The mixture was then applied to RAW264.7 cells, which were adhered to culture dish, and incubated for 6 h (37 °C, 5% CO_2). The cells were washed extensively with PBS (1 x) three times, stained with DAPI and visualized by confocal laser scanning microscopy (Zeiss LSM 710, Germany). For contrast group, all the procedures were the same as described above except the absence of virions. Besides, the DID-labeled virus was purified by Sephadex SEC (spin column, Roche; 1,100 g × 4 min) instead, and the procedures were the same except that the incubation lasted for 4 h. For negative control, the pseudovirus was replaced by that digested by PNGase F enzyme, for the imaging of which Leica SP8 was used.

To test the infectivity of the pseudovirus after nanoMIP treatment and macrophage uptake, the nanoMIP and SARS-CoV-2 pseudovirus were incubated with RAW264.7 cells in a similar way as described above. Then HEK293T-ACE2 cells were added, co-culture was carried out in a macrophage to 293T cell ratio of 1:1 for 24 h (37 °C, 5% CO_2). Confocal images were obtained as described above.

References

[1] A. Brinkø, C. Risinger, A. Lambert, O. Blixt, C. Grandjean, H. H. Jensen, *Org. Lett.* **2019**, *21*, 7544-7548.

[2] S. Qiu, W. Zhang, G. Sun, Z. Wang, J. Zhang, *ChemistrySelect*. **2016**, *1*, 4840-4844.

[3] M. Abellán Flos, M. I. García Moreno, C. Ortiz Mellet, J. M. García Fernández, J.-F. Nierengarten, S. P. Vincent, *Chem. Eur. J.* **2016**, *22*, 11450-11460.

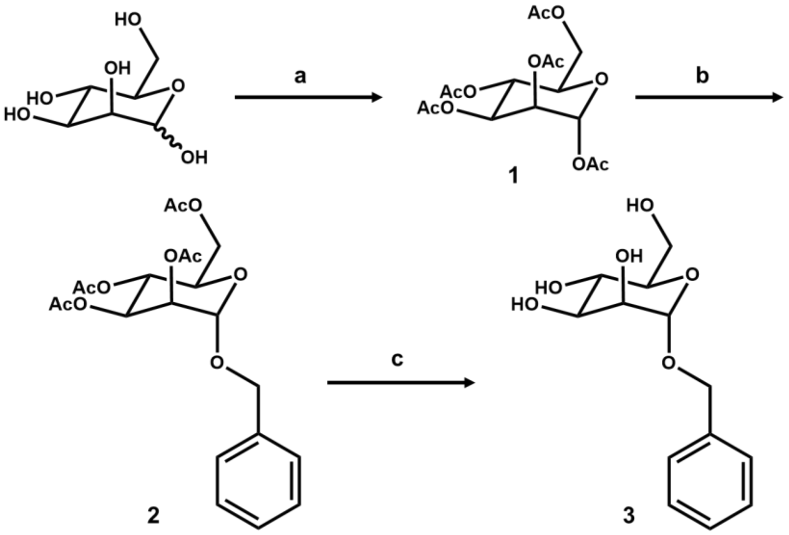

Figure S1. Synthesis of amphiphilic template Man-Bn. *Reagents and conditions*: a) Ac_2O, Py, N_2, rt., 24 h; b) BnOH, $FeCl_3$, $ClCH_2CH_2Cl$, Na_2SO_4, N_2, rt., 8 h; c) NaOMe, MeOH, N2, rt., 6 h.

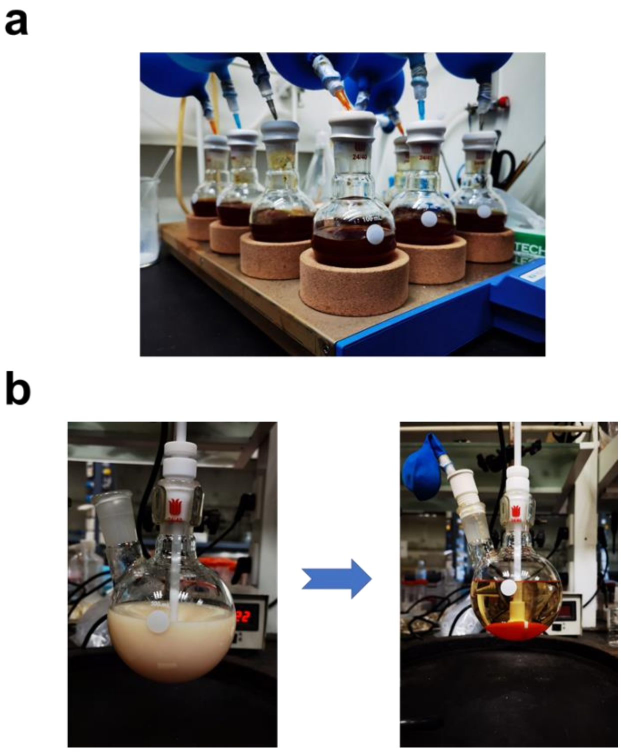

Figure S2. Photos of synthesis of amphiphilic template Man-Bn. a) Photo of mass synthesis of 2-benzyl-2,3,4,6-tetra-O-acetyl-α-D-mannopyranoside 2. b) Photos of synthesis of 2-benzyl-α-D-mannopyranoside (Man-Bn). Before (left) and after (right) sodium methoxide being neutralized.

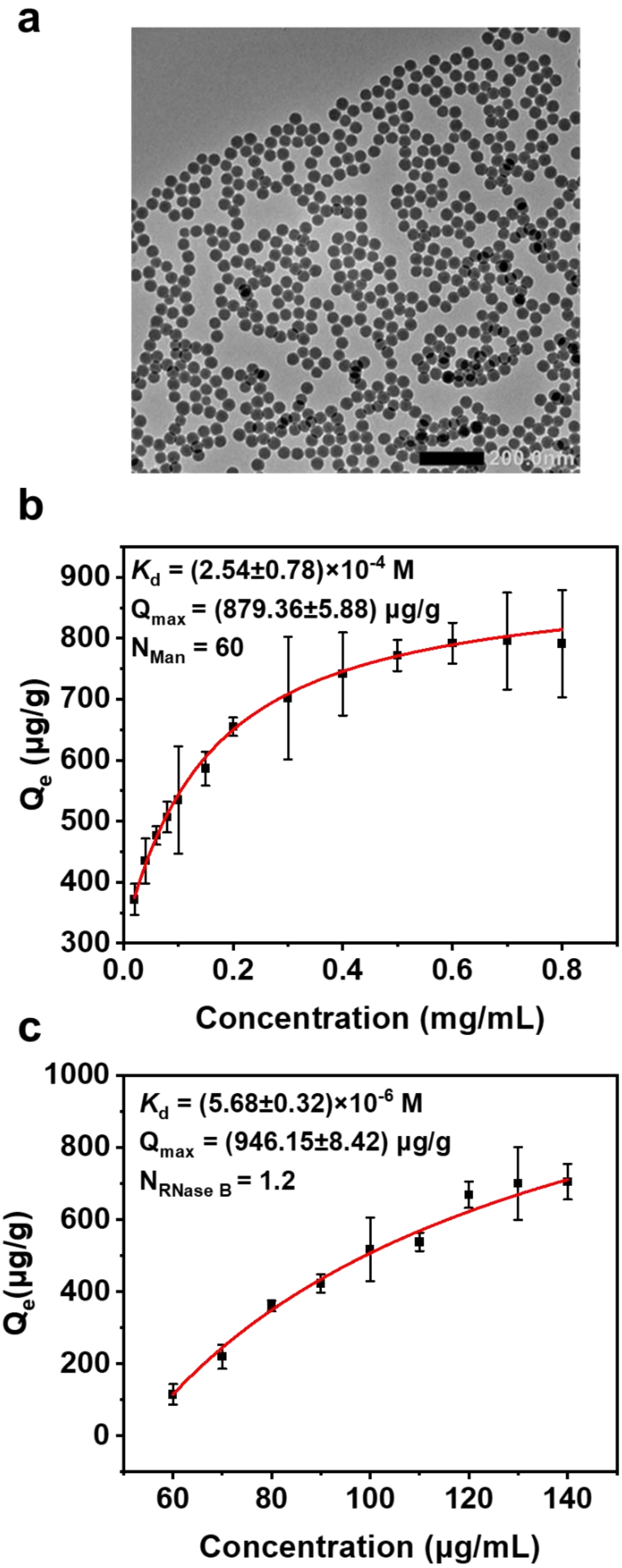

Figure S3. Characterization of Man-imprinted nanoparticles (template amount used: 1 mg). a) TEM image of Man-imprinted nanoparticles. b) and c) Adsorption isotherms for the binding of Man-imprinted nanoparticles towards Man-Bn and RNase B. Mean ± SD, n = 3.

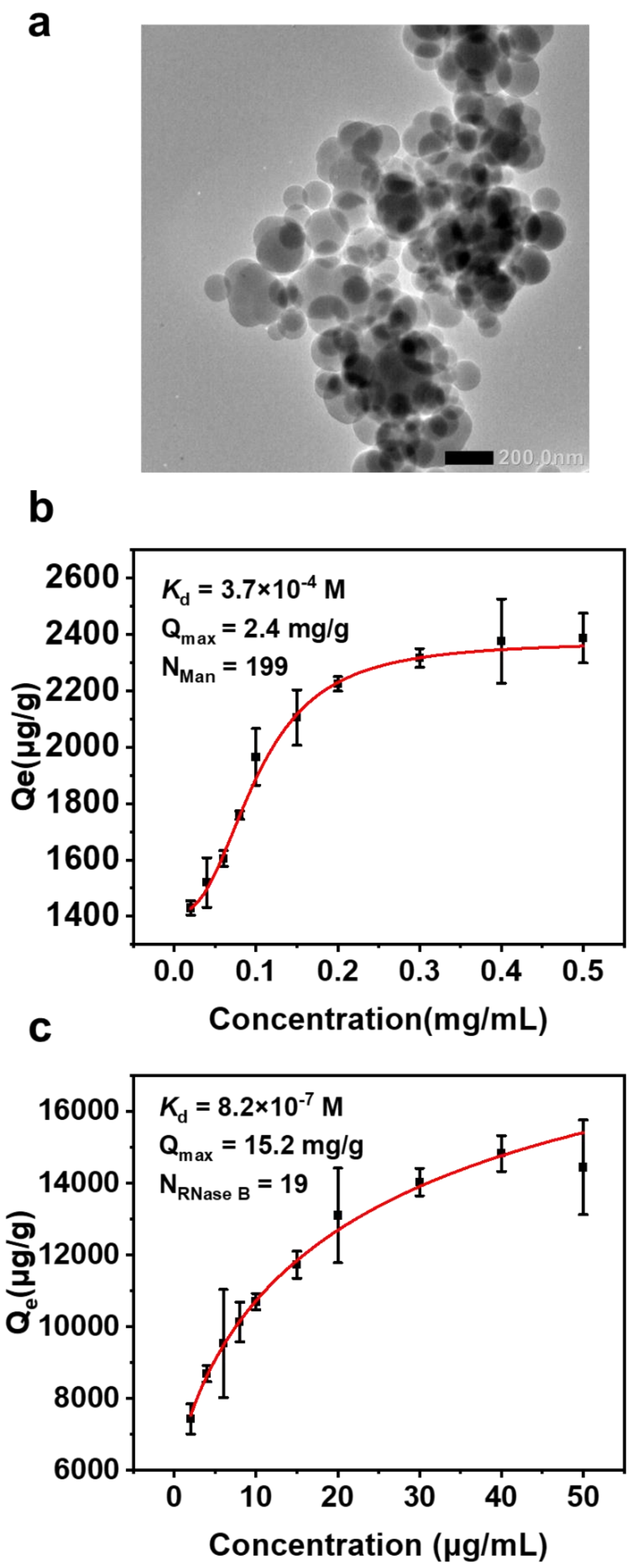

Figure S4. Characterization of Man-imprinted nanoparticles (template amount used : 1 g). a) TEM image of Man-imprinted nanoparticles. b) and c) Adsorption isotherms for the binding of Man-imprinted nanoparticles towards Man-Bn and RNase B. Mean ± SD, n = 3.

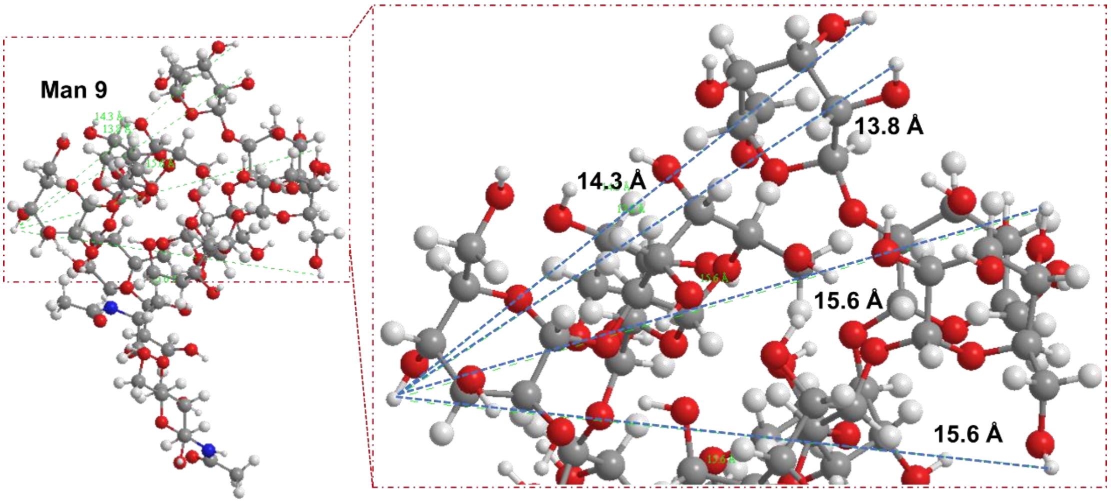

Figure S5. Distance between different branches of high mannose glycan (Man 9) measured by Chem3D 19.0 software.

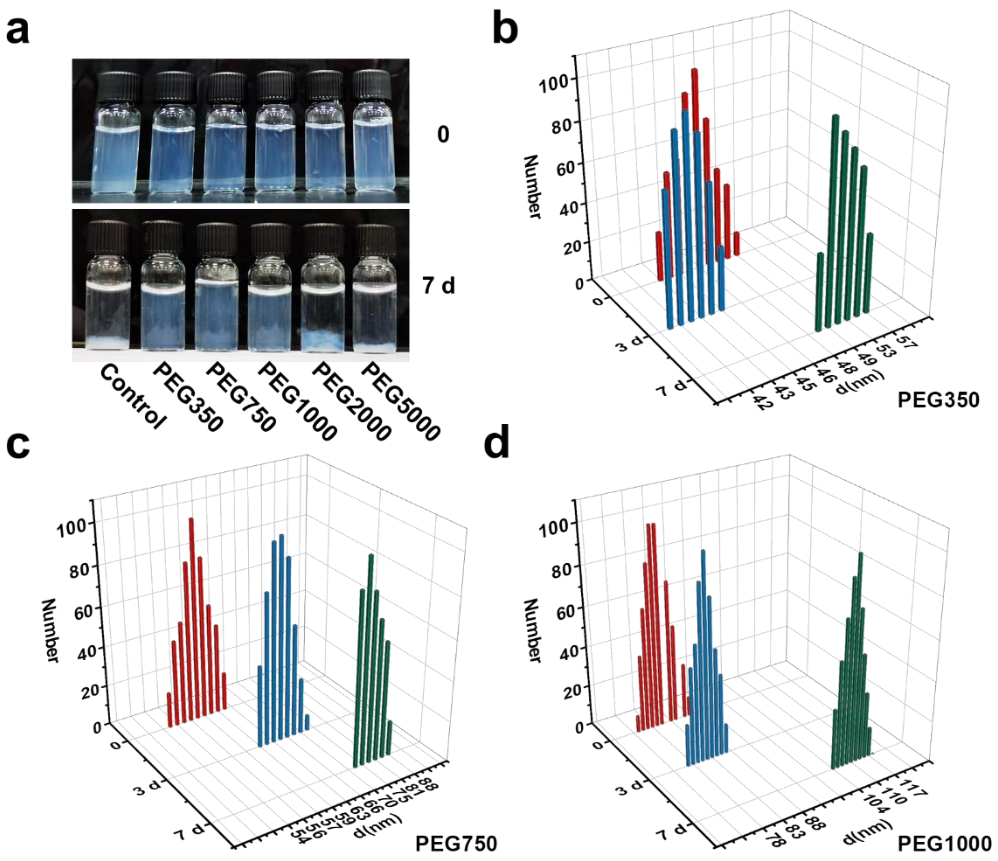

Figure S6. Optimization of PEG chain length via *in vitro* stability. a) Images of nanoMIP in PBS (1 x) before and after storing for 7 days. b-d) DLS characterization of nanoMIP surface modified with PEG chains (350 Da, 750 Da, 1000 Da) after storing for 0, 3 and 7 days.

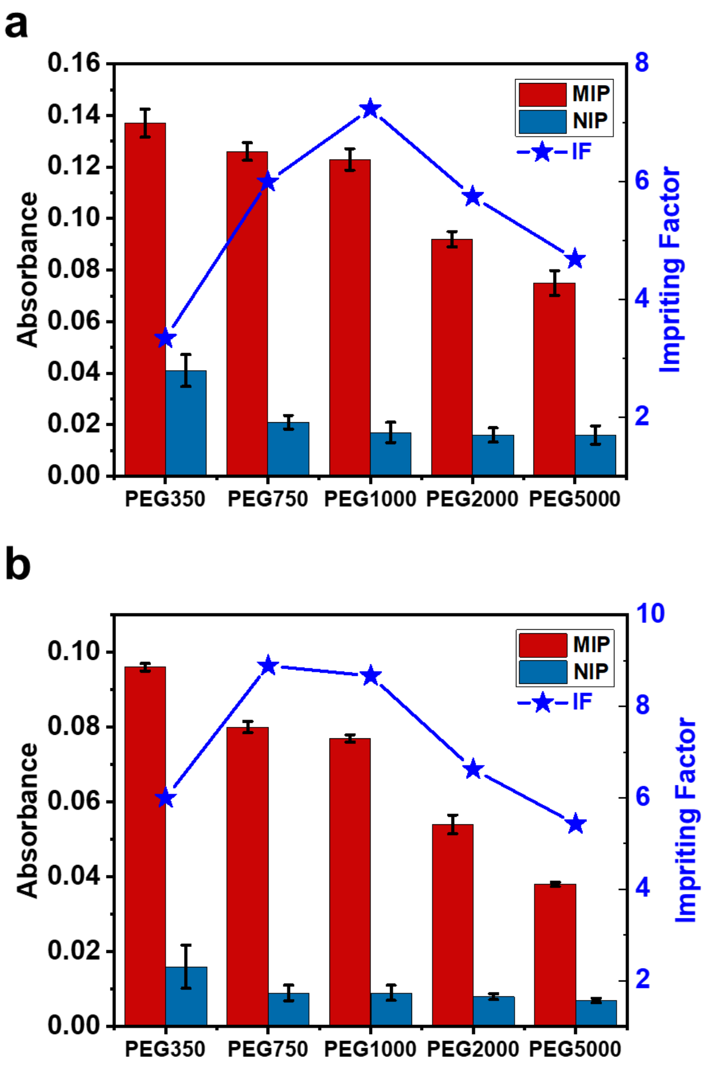

Figure S7. Optimization of PEG chain length in terms of the binding capability towards Man-Bn (a) and RNase B (b) and the corresponding imprinting factor. Mean ± SD, n = 3.

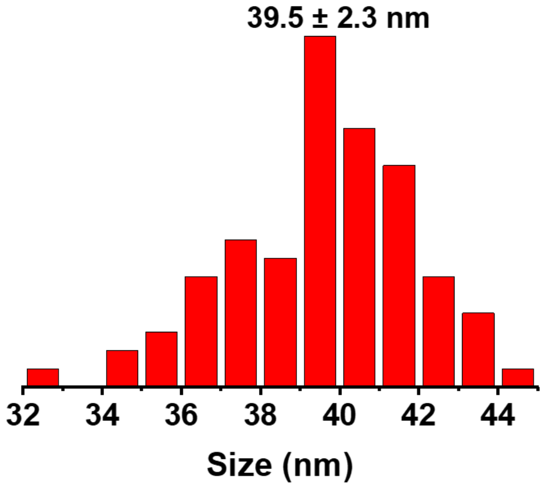

Figure S8. The size distribution of nanoMIP by Image J analysis of TEM images.

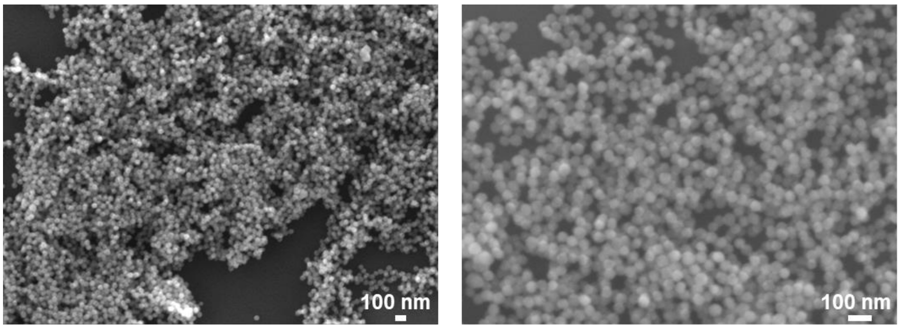

Figure S9. SEM images of nanoMIP.

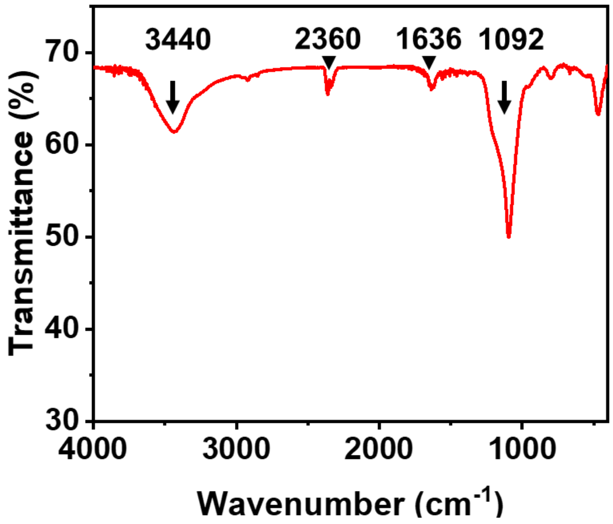

Figure S10. FT-IR spectrum for nanoMIP.

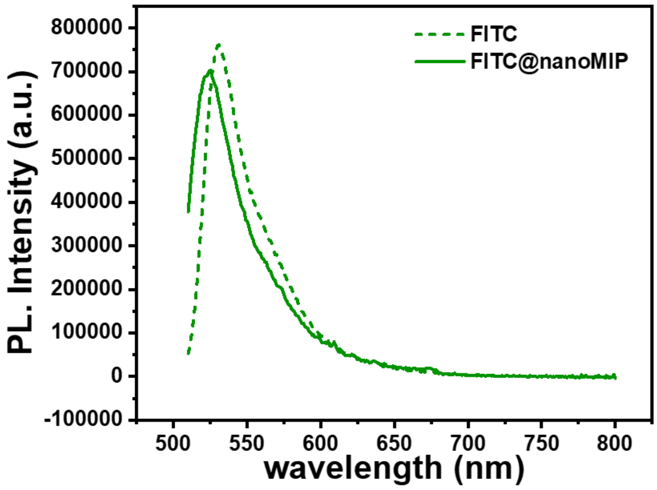

Figure S11. Fluorescence spectra for FITC and FITC doped nanoMIP.

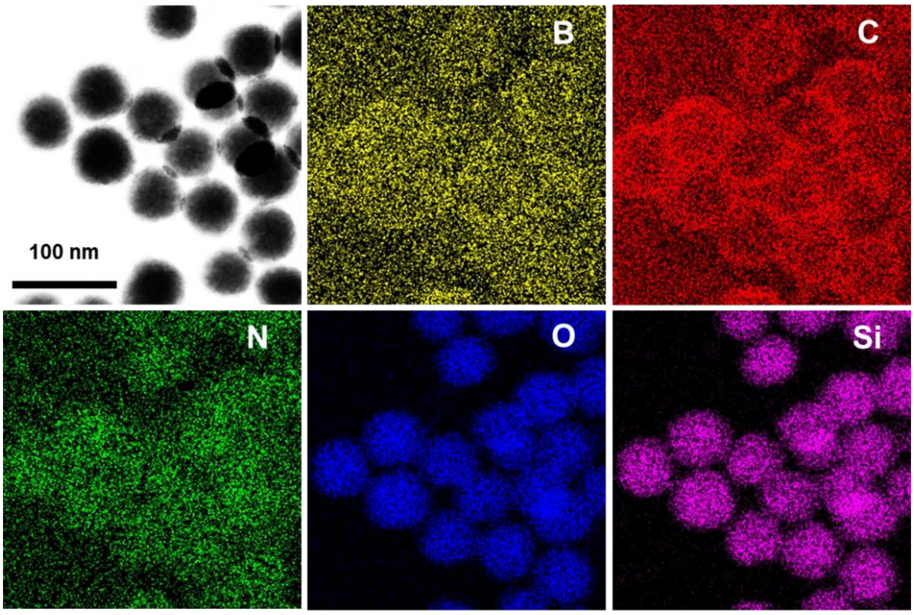

Figure S12. STEM image and EDS mapping of nanoMIP.

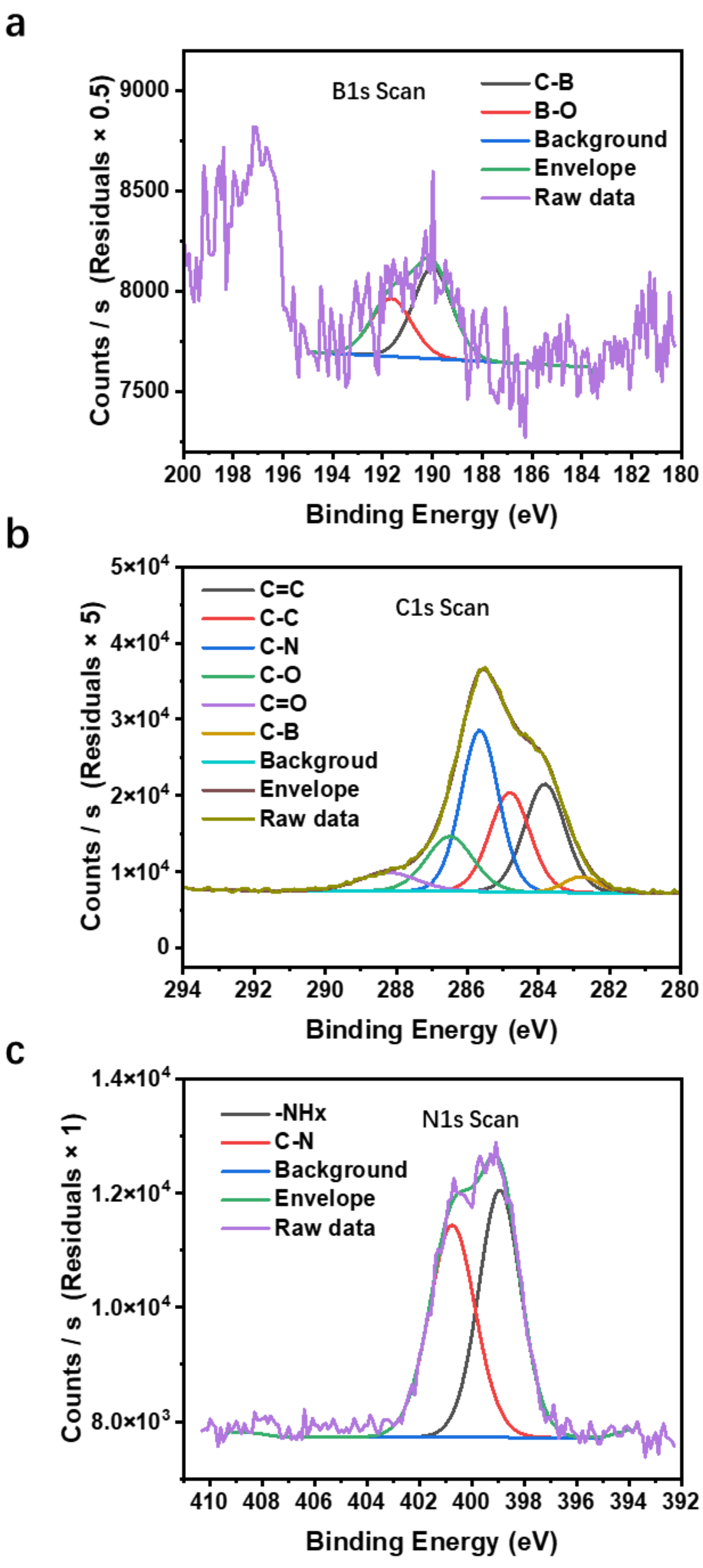
a
B1s Scan
C-B
B-O
Background
Envelope
Raw data
Counts / s (Residuals × 0.5)
9000
8500
8000
7500
200 198 196 194 192 190 188 186 184 182 180
Binding Energy (eV)
b
C1s Scan
C=C
C-C
C-N
C-O
C=O
C-B
Backgroud
Envelope
Raw data
Counts / s (Residuals × 5)
5×10^4
4×10^4
3×10^4
2×10^4
1×10^4
0
294 292 290 288 286 284 282 280
Binding Energy (eV)
c
N1s Scan
-NHx
C-N
Background
Envelope
Raw data
Counts / s (Residuals × 1)
1.4×10^4
1.2×10^4
1.0×10^4
8.0×10^3
410 408 406 404 402 400 398 396 394 392
Binding Energy (eV)

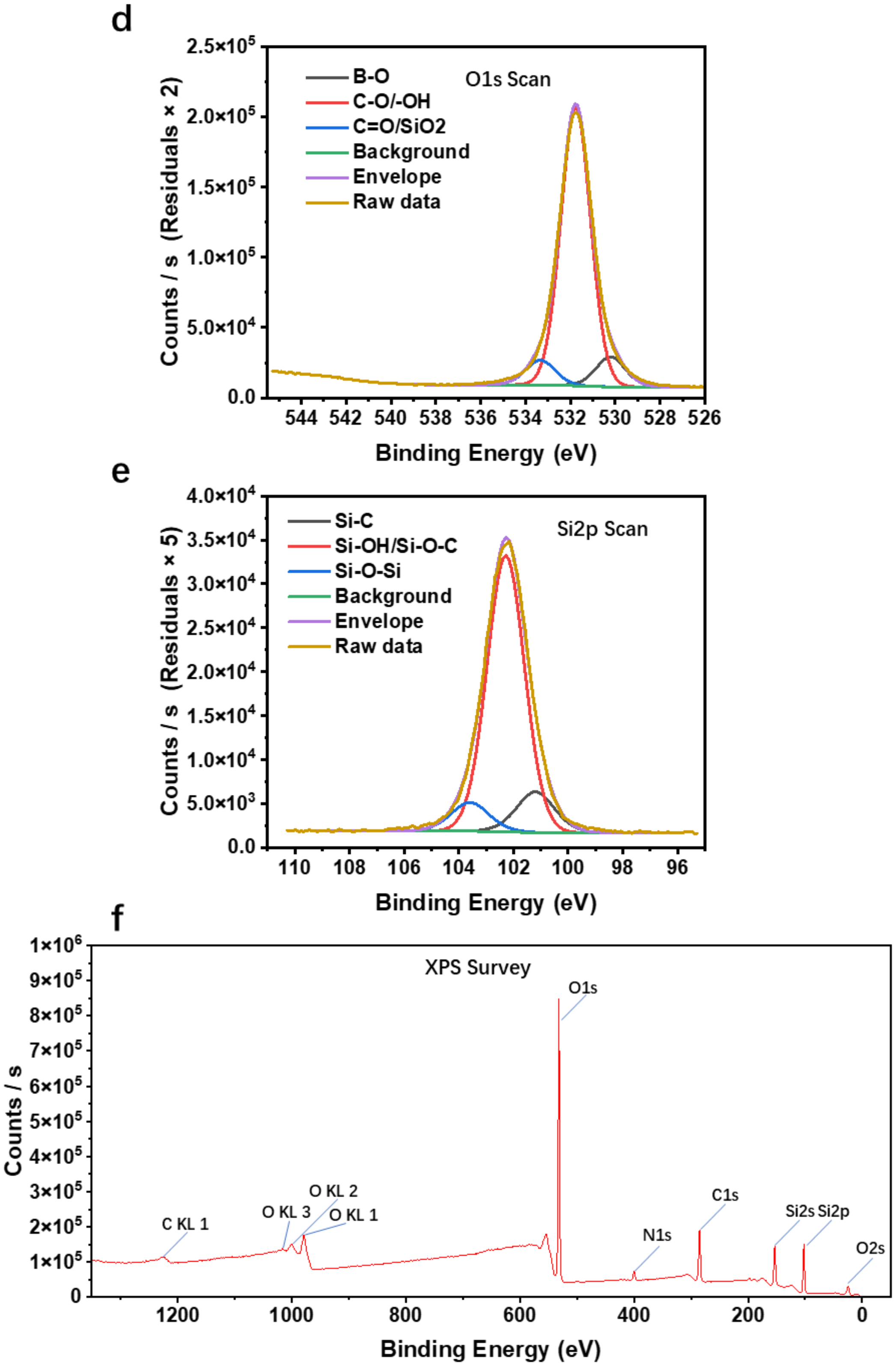

Figure S13. The XPS analyses of nanoMIP. Detail scan (a-e) and XPS survey (f).

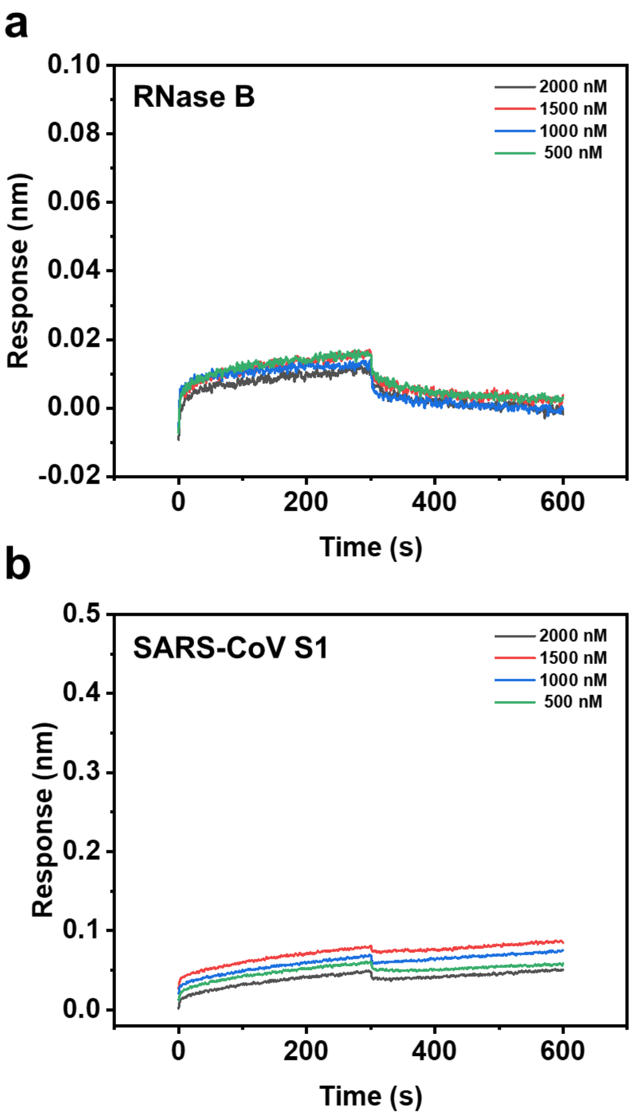

Figure S14. Binding affinity measurement of NIP. The binding curves of NIP towards RNase B (a) and SARS-CoV-2 S1(b).

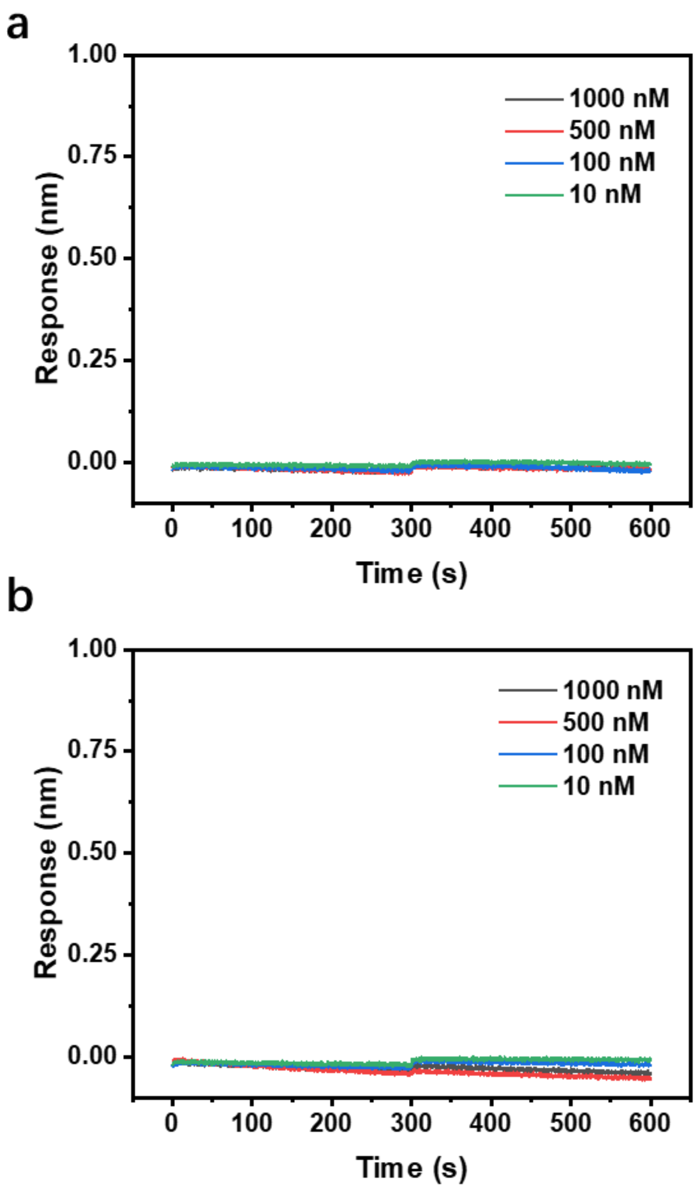

Figure S15. Binding affinity control experiments of nanoMIP. a) The binding curves of pseudovirus without N-glycans towards nanoMIP. b) The binding curves of pseudovirus towards nanoMIP pre-incubated with 10 mg/mL RNase B solution. SARS-CoV-2 pseudovirus, wild type.

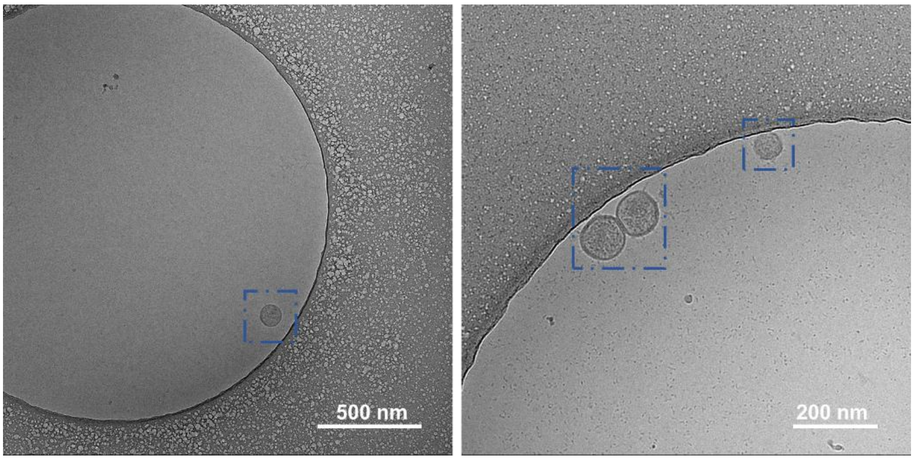

Figure S16. Cryo-TEM images of SARS-CoV-2 pseudovirus (wild type). The virus was marked by blue dashed box.

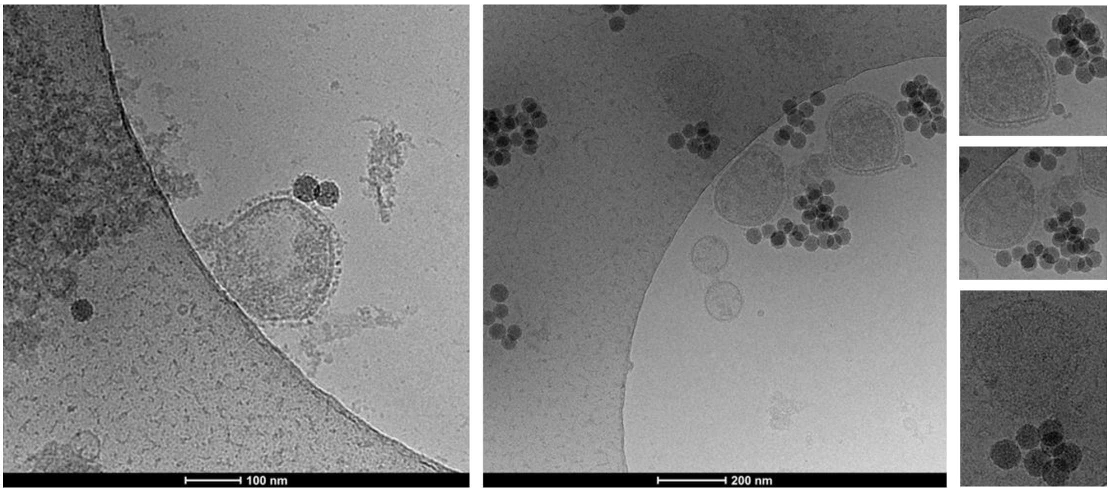

Figure S17. Cryo-transmission electron microscopy images for the SARS-CoV-2 pseudovirus particles binding with nanoMIP (25 μg/mL). Small images were cropped from origin image and highlighted.

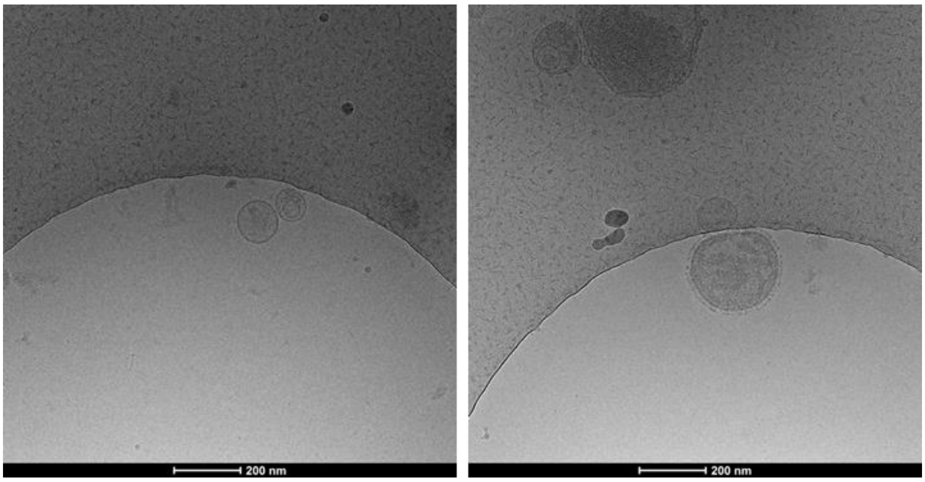

Figure S18. Cryo-transmission electron microscopy images for the SARS-CoV-2 pseudovirus particles treated with NIP (25 μg/mL).

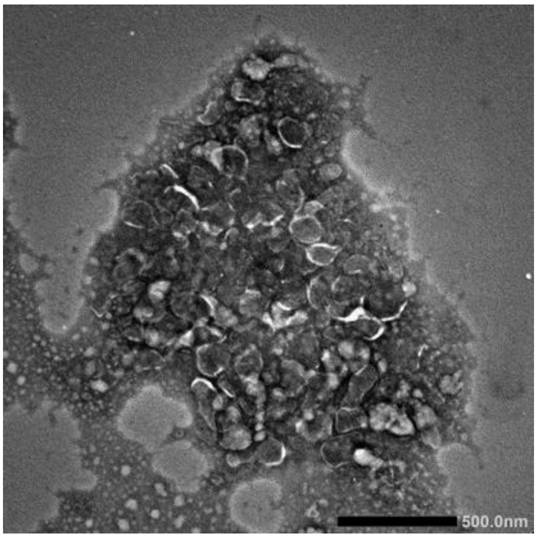

Figure S19. Negatively stained transmission electron microscopy images for virus aggregates. Scale bar, 500 nm.

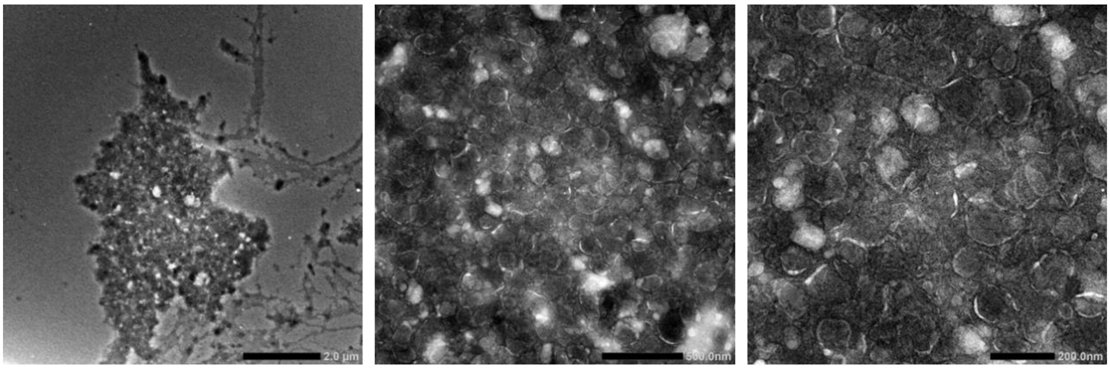

Figure S20. Negatively stained transmission electron microscopy images for virus aggregates. The same aera was gradually zoomed in with scale bars from 2 μm, 500 nm to 200 nm, respectively.

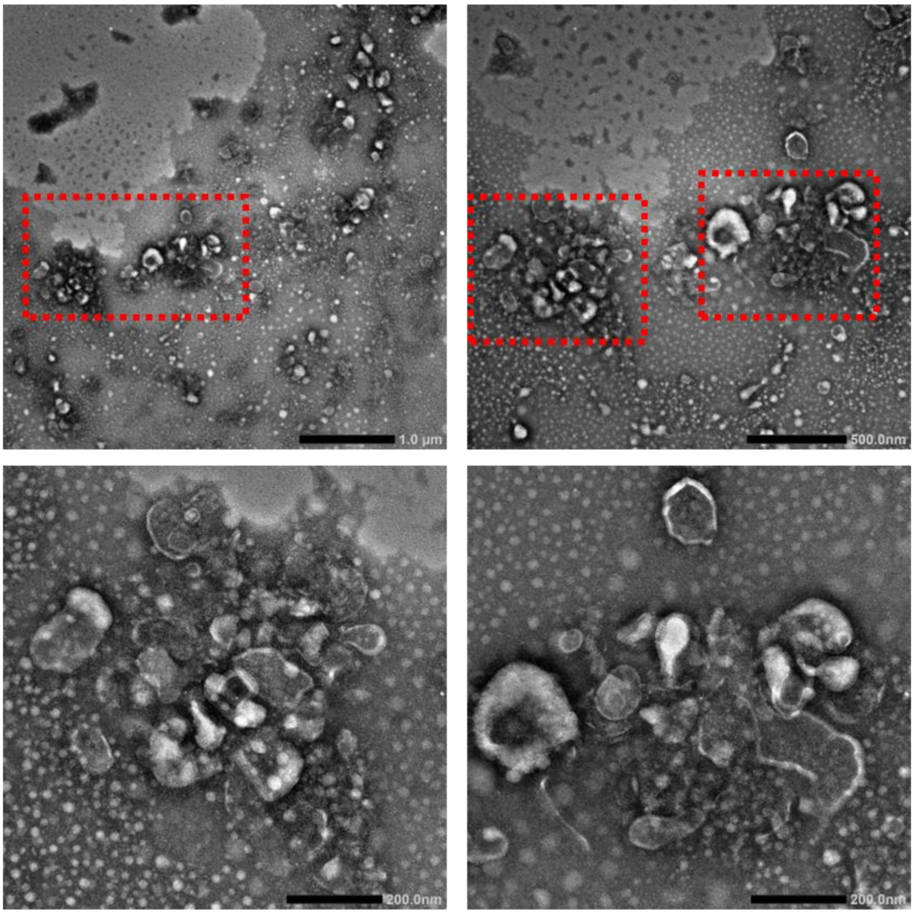

Figure S21 Negatively stained transmission electron microscopy images for virus aggregates. The red dotted box is the enlarged area. Scale bars, 1 μm, 500 nm and 200 nm, respectively.

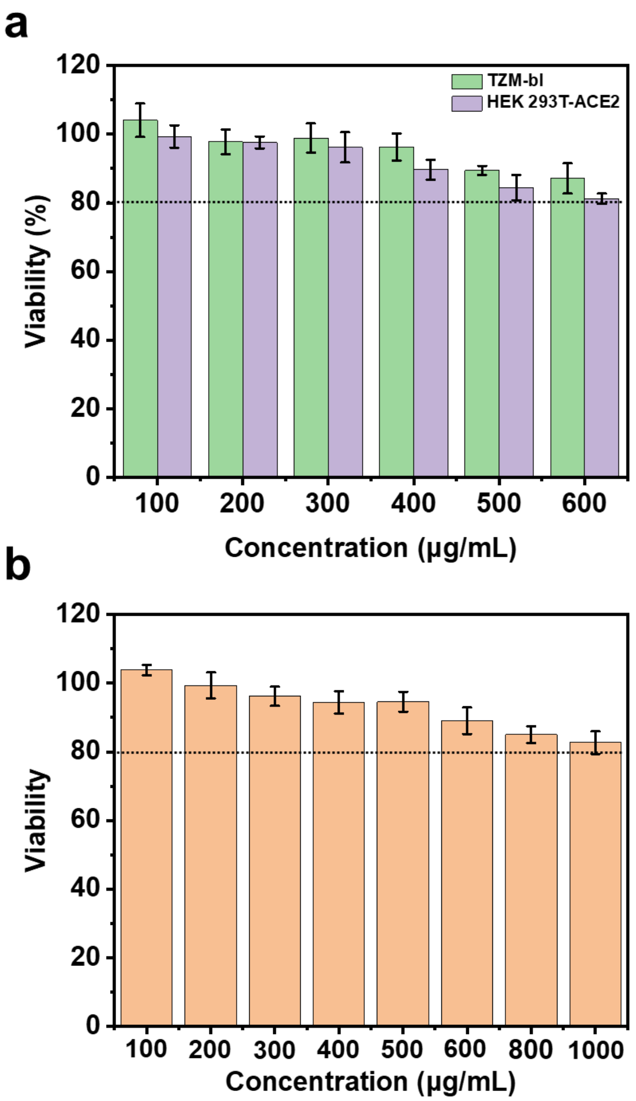

Figure S22. a) Cell viability of TZM-bl and HEK293T-ACE2 cells treated with different concentrations of nanoMIP. b) Cell viability of Vero cells treated with different concentrations of nanoMIP. Mean ± SD, n = 3.

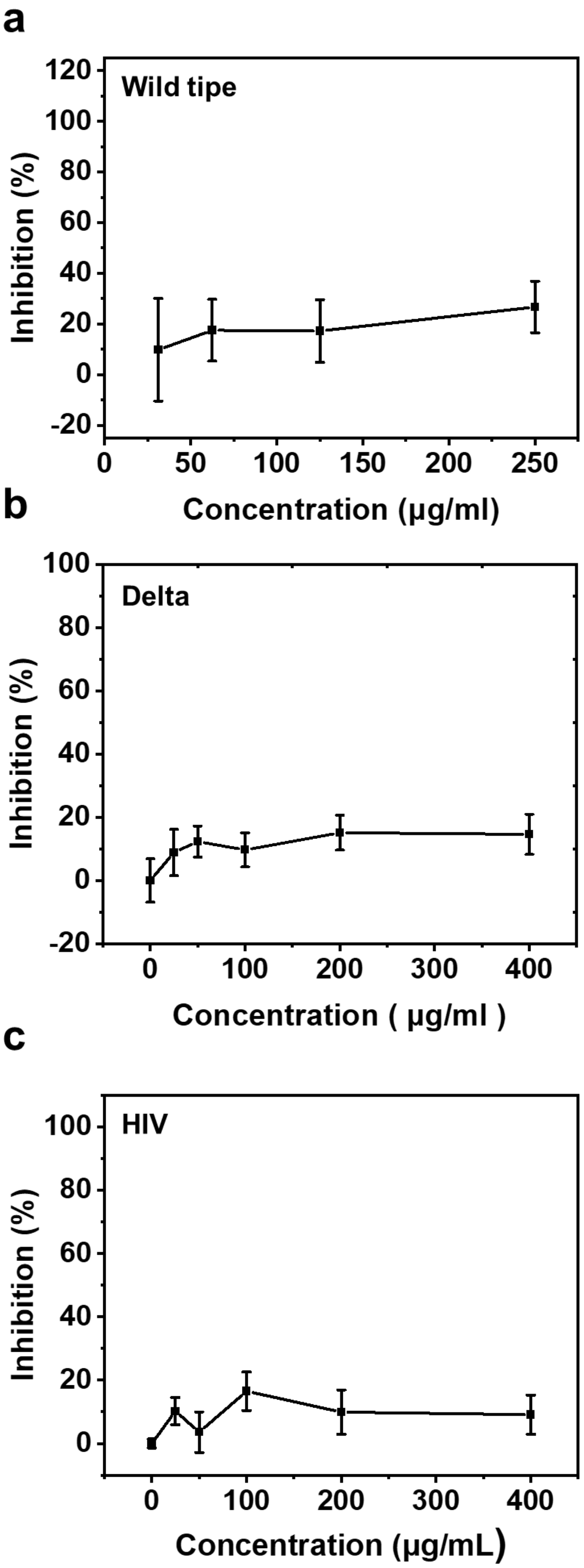

Figure S23. Inhibition of different pseudoviruses infectivity by NIP for contrast. a) SARS-CoV-2 pseudovirus (wild type). b) SARS-CoV-2 pseudovirus (delta). c) HIV pseudovirus. Mean ± SD, n = 3.

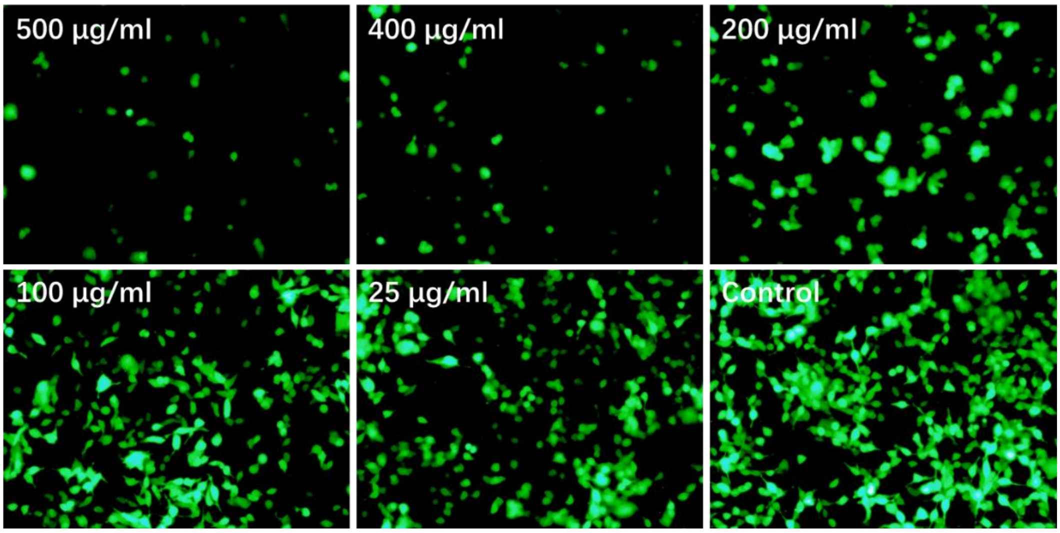

Figure S24. Representative inverted fluorescence microscopy images of ACE2-HEK293T cells without or with different concentrations of nanoMIP under the infection of SARS-CoV-2 pseudovirus (wild type) for 48 h. Green represents the fluorescent proteins in the cells.

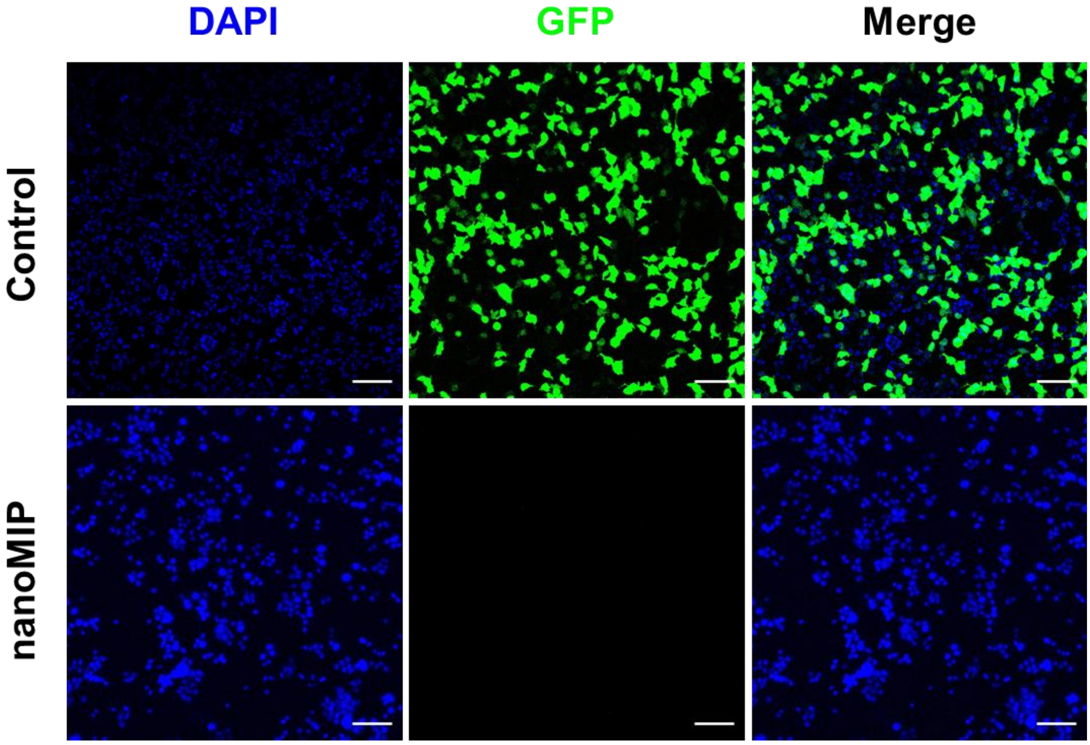

Figure S25. Representative confocal laser scanning microscopy images of ACE2-HEK293T cells without or with treatment under the infection of SARS-CoV-2 pseudovirus for 48 h. Scale bars, 100 µm.

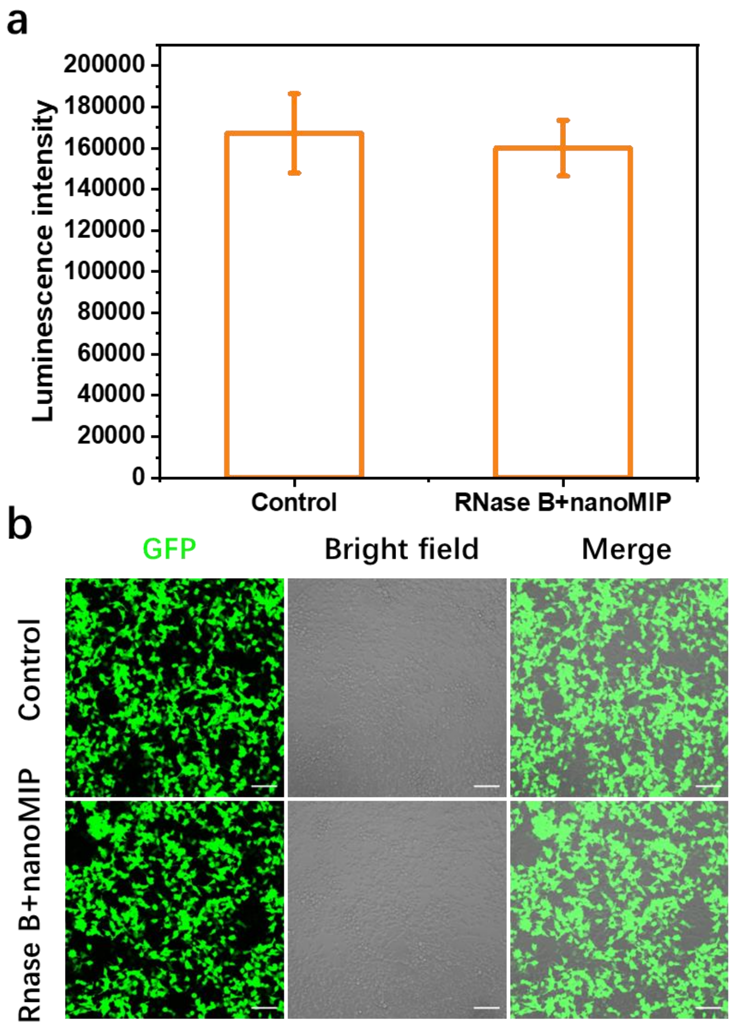

Figure S26. Luciferase luminescence intensity (a) and confocal laser scanning microscopy images (b) of ACE2-HEK293T cells infected by SARS-CoV-2 pseudovirus treated with or without 400 μg/mL nanoMIP (pre-incubated with 10 mg/mL RNase B solution). Scale bar, 100 μm. Mean ± SD, n = 3.

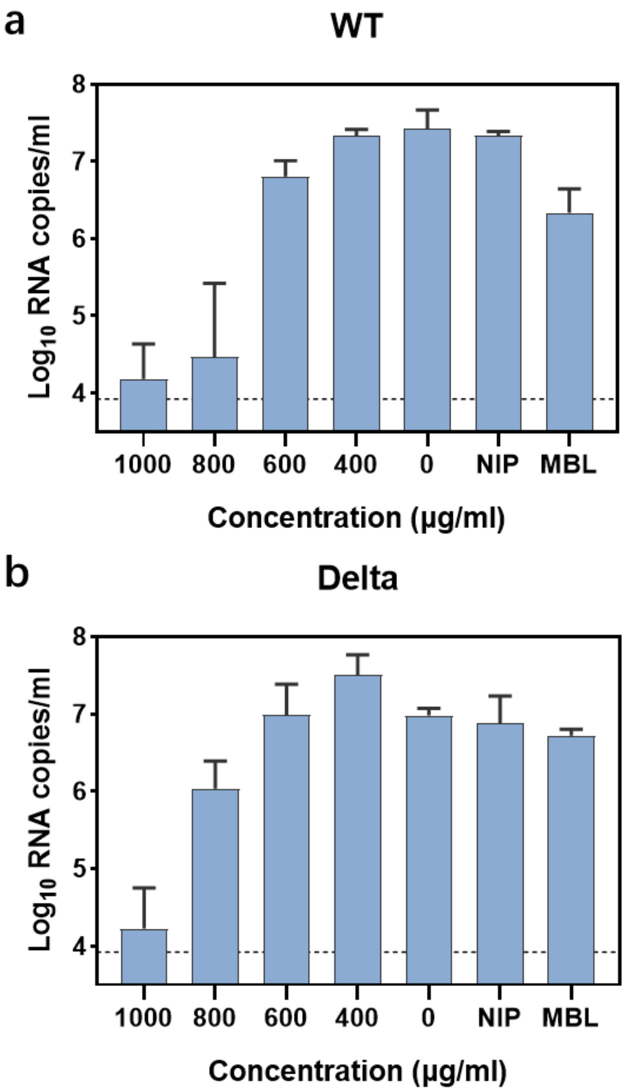

Figure S27. Quantification of authentic SARS-CoV-2 subgenomic RNA (sgRNA). a) Wild type. b) Delta strain. Mean ± SD, n = 3.

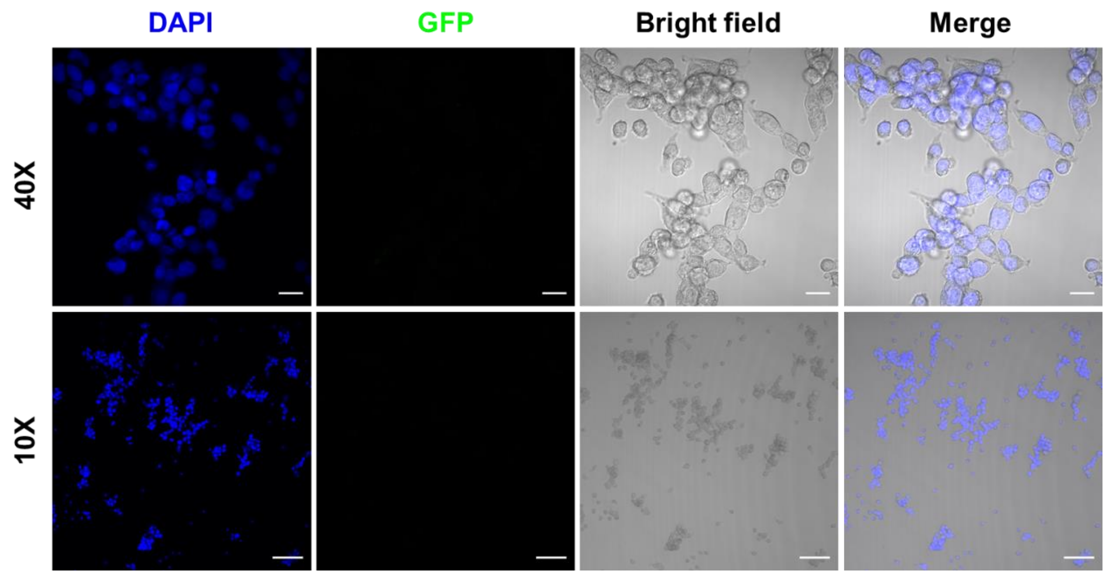

Figure S28. Confocal laser scanning microscopy images for the virus infection after nanoMIP treatment and macrophage uptake. Scale bars, 40X, 20 μm; 10X, 100 μm.

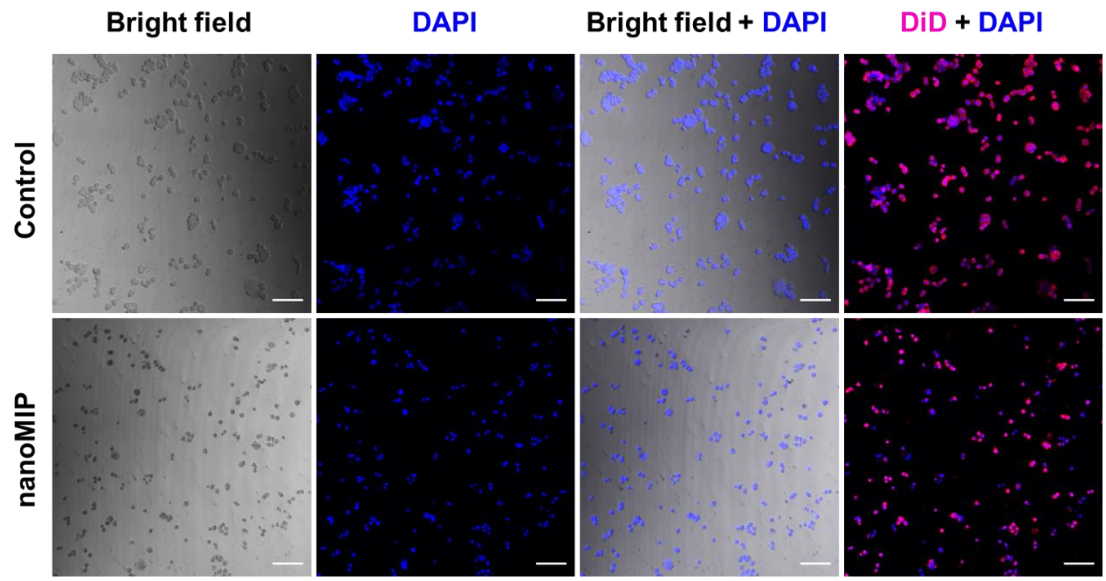

Figure S29. Confocal laser scanning microscopy images for the formation of syncytium in ACE2-HEK293T cells 24 h after SARS-CoV-2 infection with or without nanoMIP treatment. Scale bars, 100 μm.

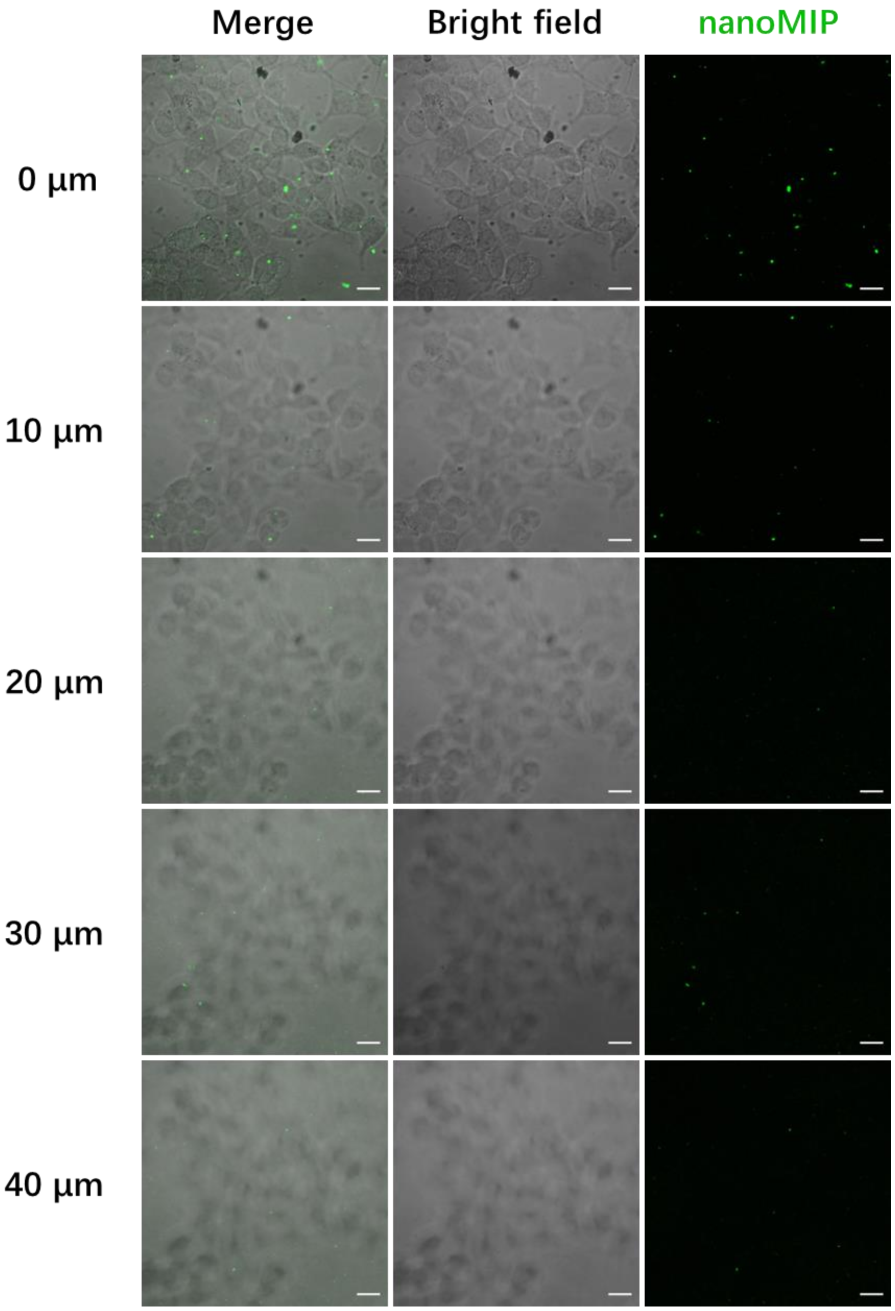

Figure S30. Confocal fluorescence images of ACE2-HEK293T cells incubated with nanoMIP (1 mg/mL) for 1 h without washing step. We focused on a specific area of the dish and moved the stage along the z axis by 0, 10, 20, 30, 40 μm, respectively. Scale bar, 20 μm.

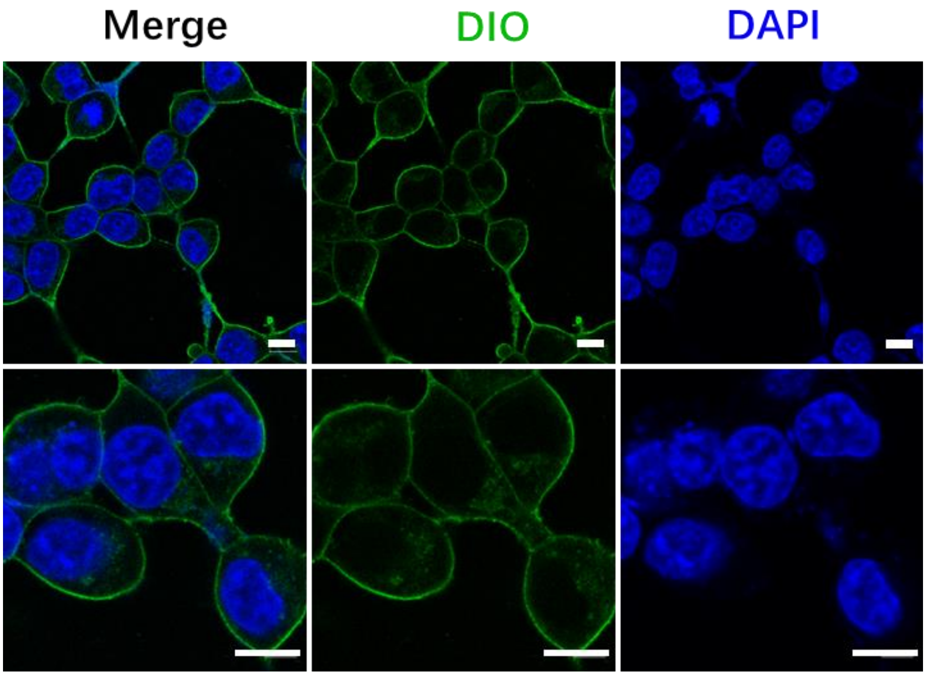

Figure S31. DIO-only control experiment. Confocal fluorescence images of ACE2-HEK293T cells stained by DIO. Green represents the cell membrane stained by DIO and blue represents the nucleus stained by DAPI. Scale bar, 10 μm.

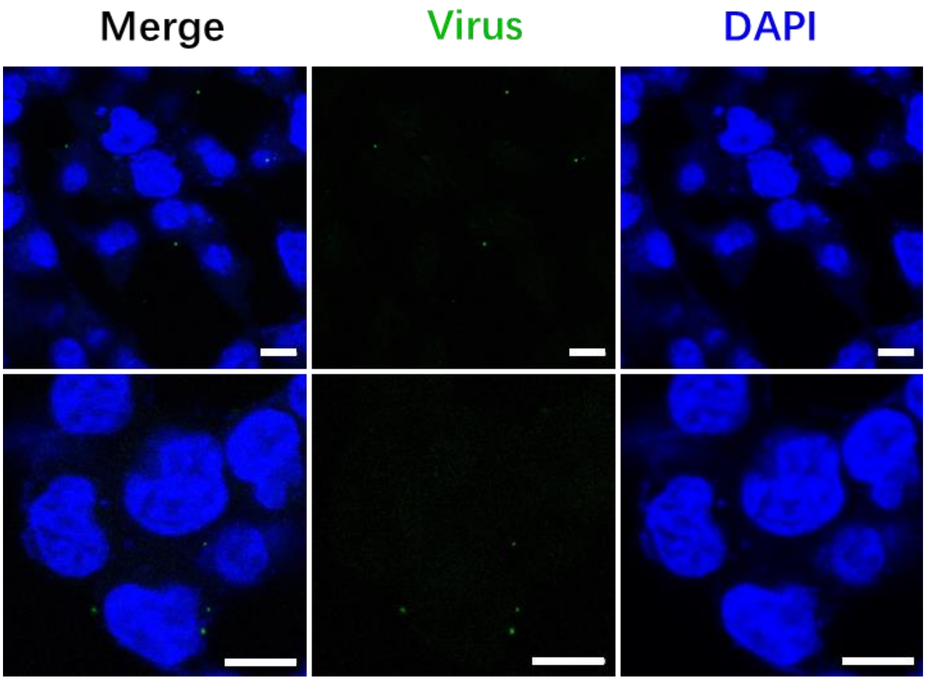

Figure S32. Confocal fluorescence images of ACE2-HEK293T cells and SARS-CoV-2 pseudovirus (All N-glycosylation were digested by PNGase F enzyme, wild type) treated with 1 mg/mL nanoMIP. Green represents the virus stained by DIO and blue represents the nucleus stained by DAPI. Scale bar, 10 μm.

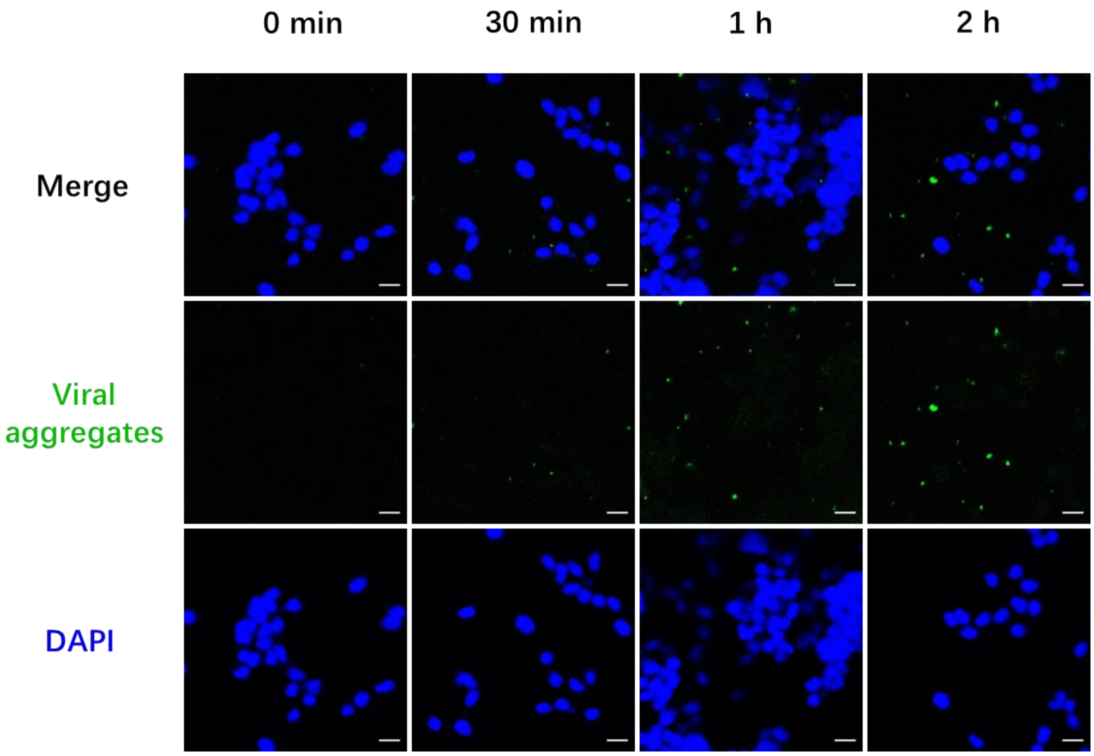

Figure S33. Confocal fluorescence images for ACE2-HEK293T cells and SARS-CoV-2 pseudovirus (wild type) treated with 200 μg/mL nanoMIP for 0, 0.5, 1 and 2 h, respectively. Green represents the virus stained by DIO and blue represents the nucleus stained by DAPI. Scale bar, 20 μm.

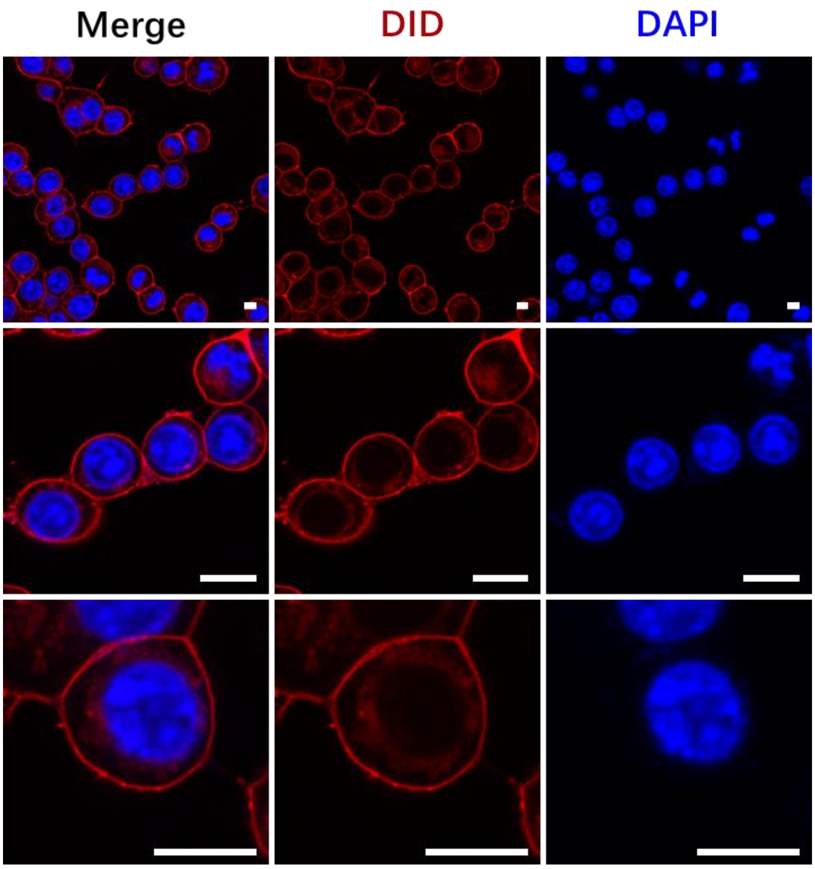

Figure S34. DID-only control experiment. Confocal fluorescence images of ACE2-HEK293T cells stained by DID. Red represents the cell membrane stained by DID and blue represents the nucleus stained by DAPI. Scale bar, 10 µm.

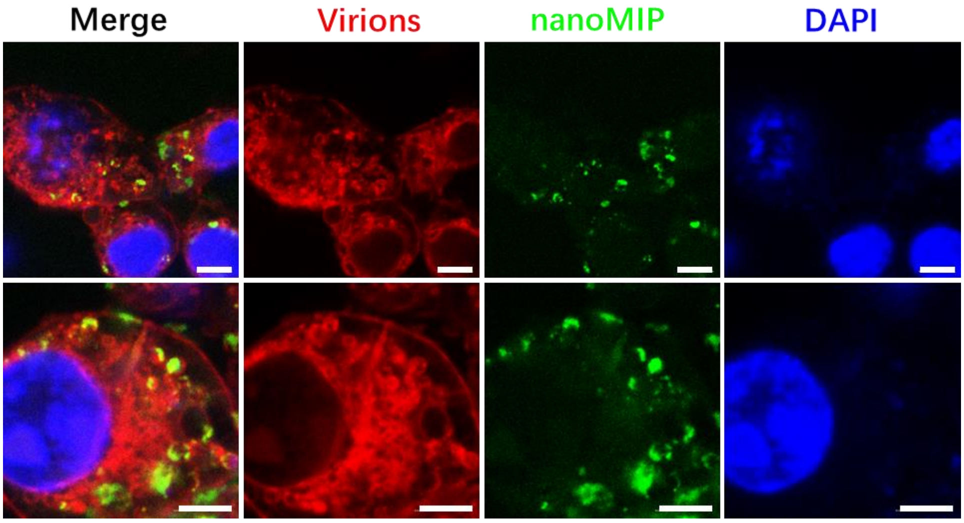

Figure S35. Confocal fluorescence images for macrophage uptake (4 h) of SARS-CoV-2 pseudovirus (wild type) treated with nanoMIP (200 µg/mL). The DID-labeled virus was purified by Sephadex SEC (spin column, Roche; 1,100 g for 4 min). Red represents the DID-labeled virions, Green represents the FITC-doped nanoMIP and blue represents the nucleus stained by DAPI. Scale bar, 5 µm.

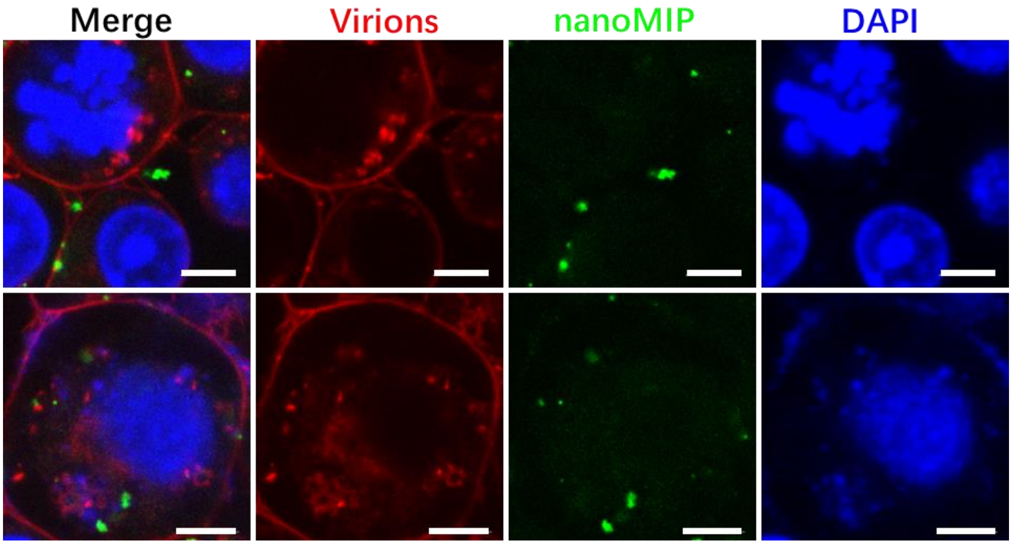

Figure S36. Confocal fluorescence images for macrophage uptake (4 h) of SARS-CoV-2 pseudovirus (All N-glycosylation were digested by PNGase F enzyme, wild type) treated with nanoMIP (200 μg/mL). The DID-labeled virus was purified by Sephadex SEC (spin column, Roche; 1,100 g for 4 min). Red represents the DID-labeled virions, Green represents the FITC-doped nanoMIP and blue represents the nucleus stained by DAPI. Scale bar, 5 μm.

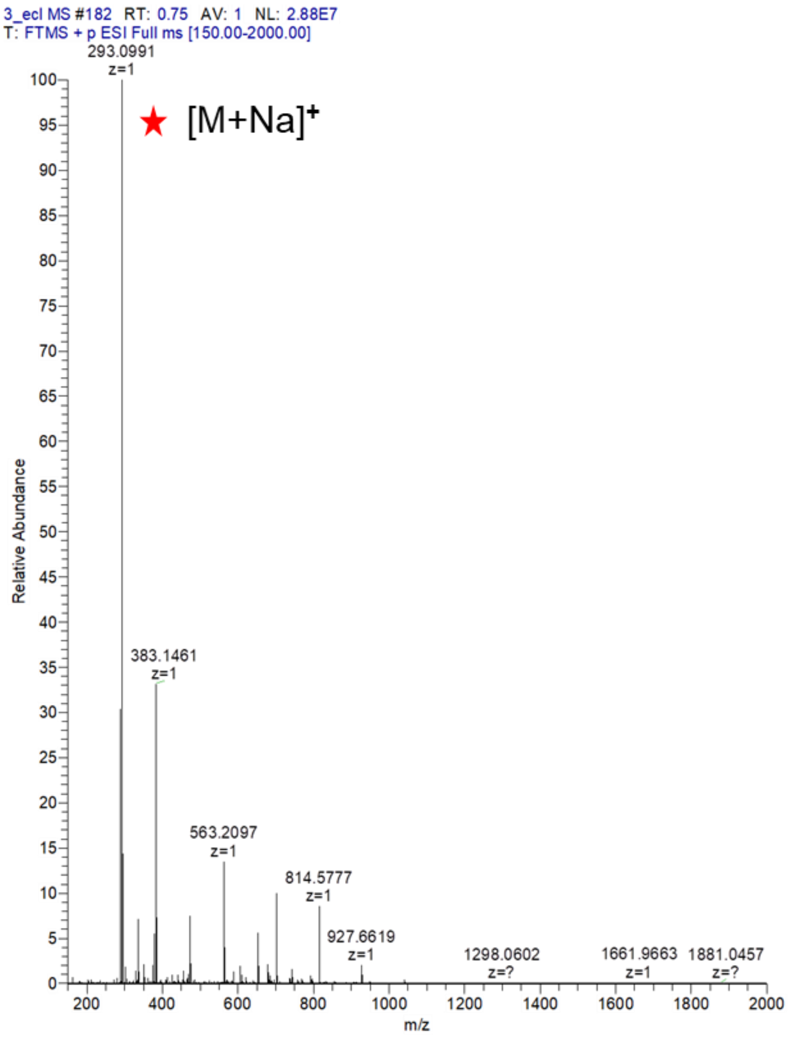

Figure S37. ESI-MS spectrum of amphiphilic template Man-Bn.

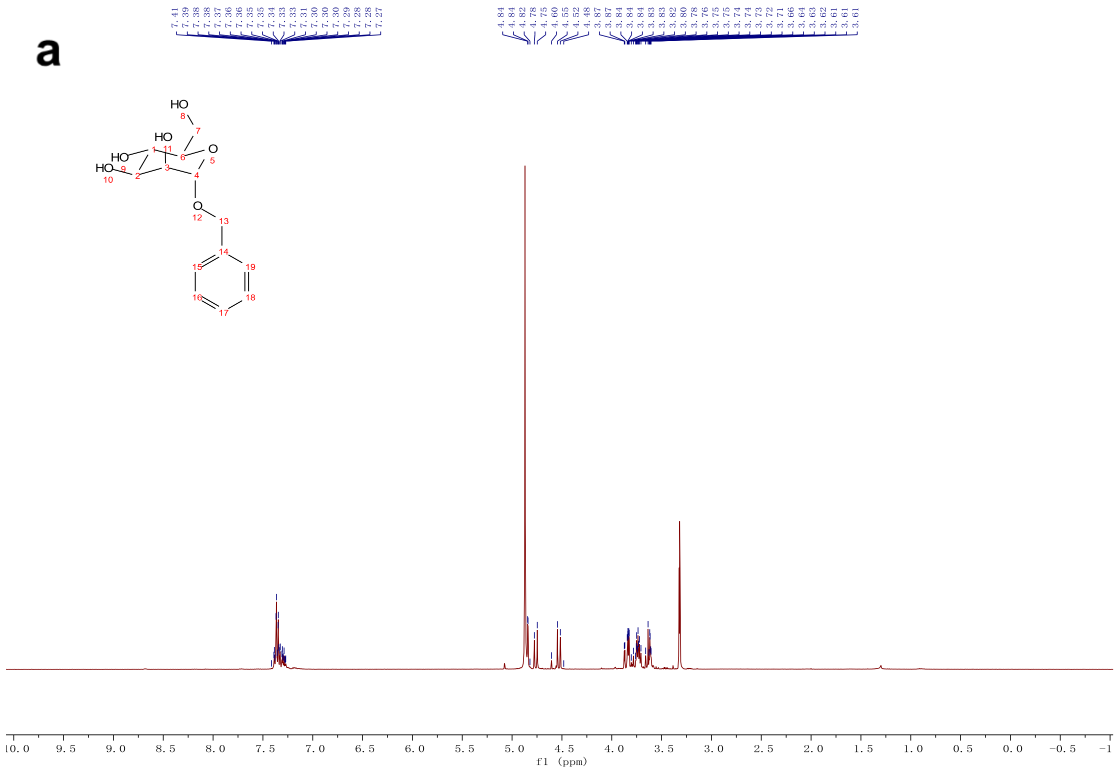

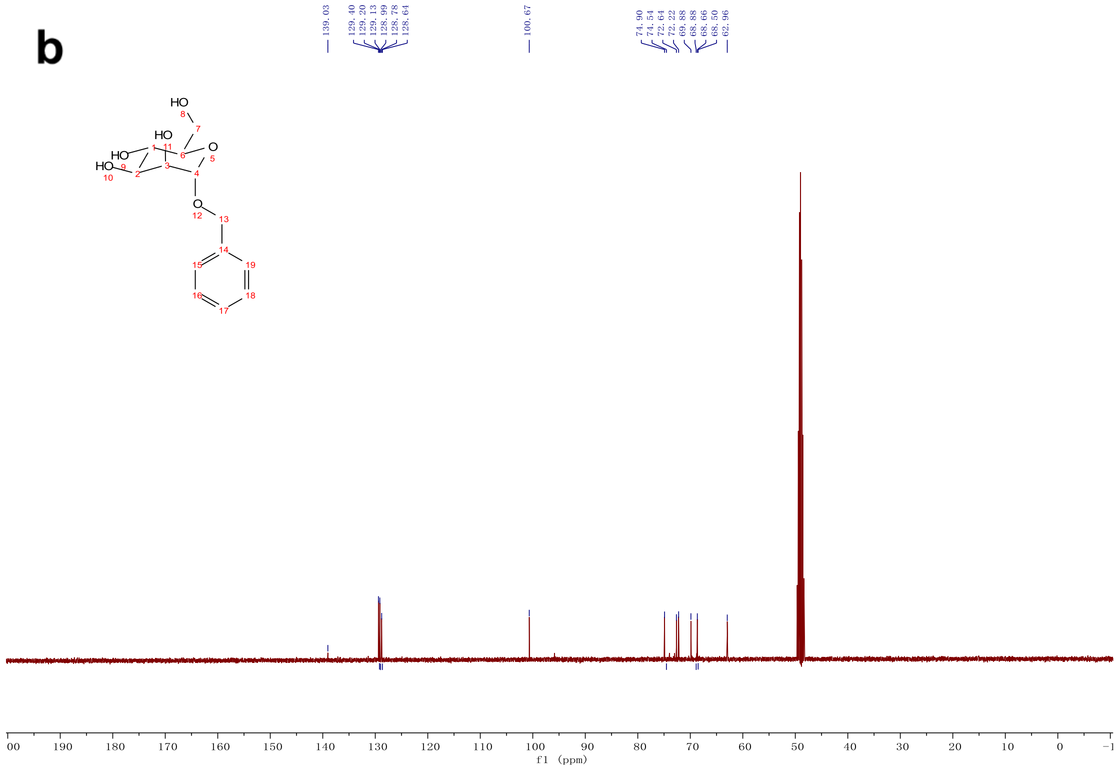

Figure S38. NMR spectra of amphiphilic template Man-Bn. a) ^{1}H NMR spectrum; b)^{13}C NMR spectrum.

a

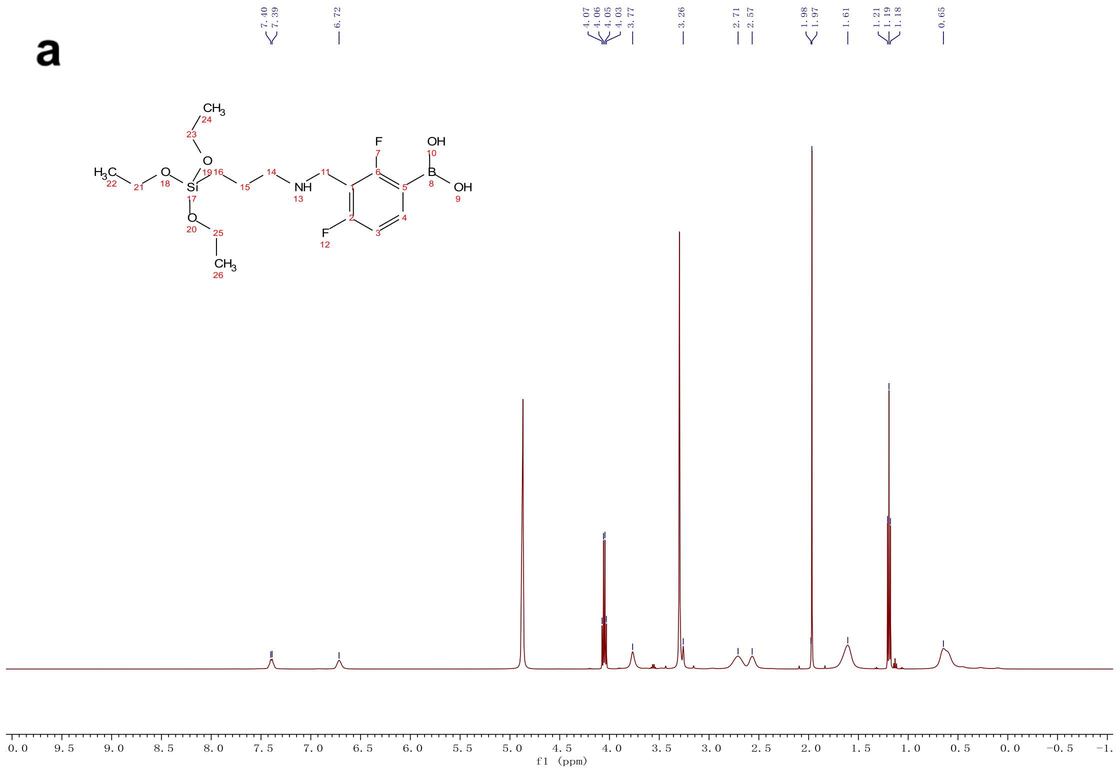

b

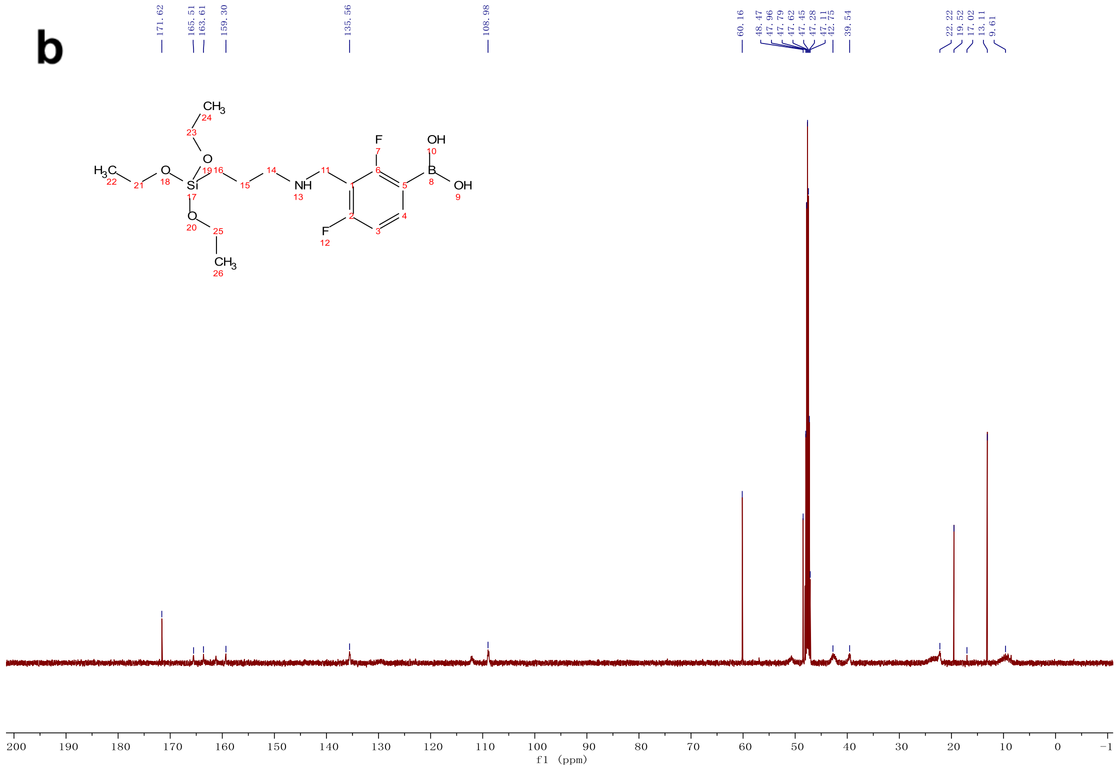

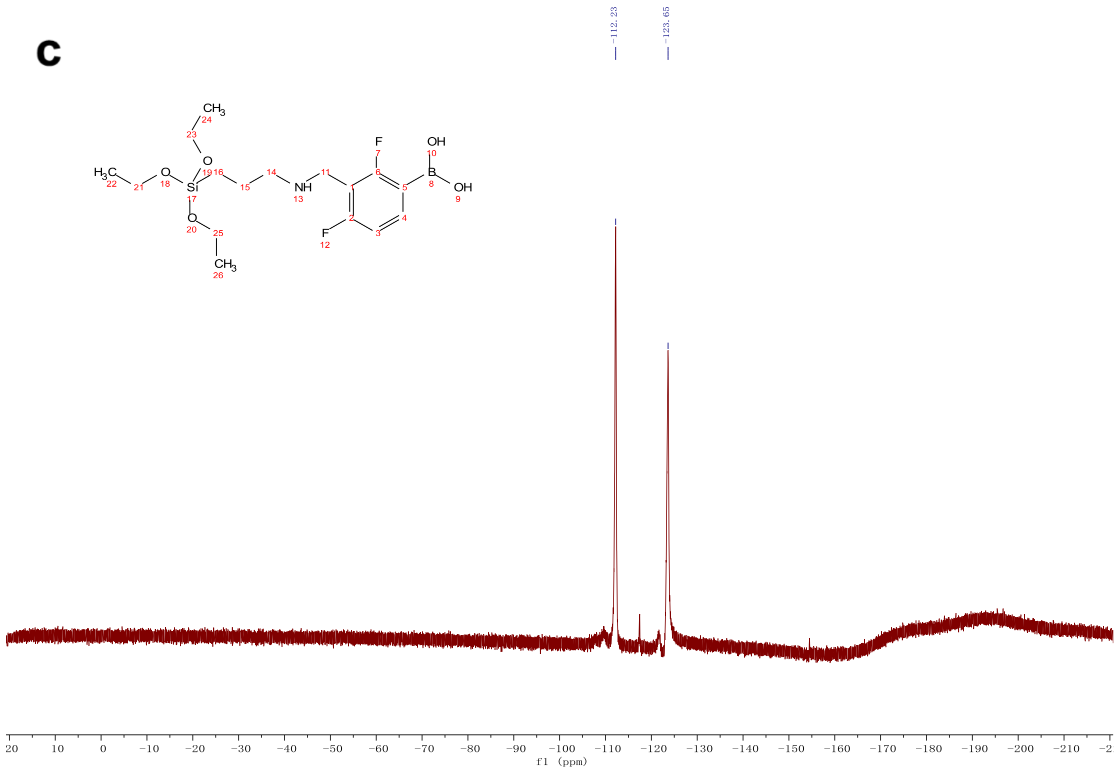

Figure S39. NMR spectra of functional monomer DFFPBA-APTES. a) ^{1}H NMR spectrum; b)^{13}C NMR spectrum; c)^{19}F NMR spectrum.

Table S1. Binding parameters for nanoMIP towards intact proteins RNase B, SARS-CoV-2 S1, HIV GP120 and SARS-CoV-2 pseudovirus by BLI.

Item	K_d / M	R^2	k_{on} / M^{-1} s^{-1}	k_{off} / s^{-1}
RNase B	1.26×10^{-6}	0.95	1.51×10^{4}	1.89×10^{-2}
SARS-CoV-2 S1	5.29×10^{-7}	0.95	1.47×10^{4}	7.79×10^{-3}
HIV GP120	5.42×10^{-7}	0.94	1.64×10^{4}	8.87×10^{-3}
SARS-CoV-2 pseudovirus	8.50×10^{-10}	0.99	2.05×10^{5}	1.74×10^{-4}
